# Parity predisposes breasts to the oncogenic action of PAPP-A and activation of the collagen receptor DDR2

**DOI:** 10.1186/s13058-019-1142-z

**Published:** 2019-05-02

**Authors:** Elizabeth Slocum, Amanda Craig, Augusto Villanueva, Doris Germain

**Affiliations:** 1 Department of Medicine, Division of Hematology/Oncology, Tisch Cancer Institute, Icahn School of Medicine at Mount Sinai, New York, NY USA; 2 Department of Medicine, Division of Liver Diseases, Liver Cancer Program, Tisch Cancer Institute, Icahn School of Medicine at Mount Sinai, New York, NY USA; 3 Graduate School of Biomedical Sciences, Icahn School of Medicine at Mount Sinai, New York, NY USA

**Keywords:** Pregnancy-associated breast cancer, DDR2, Parity, Pregnancy-associated plasma protein A, Involution, Insulin-like growth factor (IGF) signaling, Collagen, TACS, LARP6

## Abstract

**Background:**

Women who had children at a young age (less than 25) show a reduced overall risk of breast cancer. However, epidemiological studies showed that for all other women, pregnancy increases the risk of breast cancer and the risk remains higher for decades. Further, even in women who had children at a young age, there is a transient increase risk that peaks 6 years after pregnancy. Women diagnosed with breast cancer following pregnancy show a higher rate of metastasis. Yet, the factors that increase the predisposition of post-partum breasts to more aggressive cancers remain unknown. Pregnancy-associated plasma protein A (PAPP-A) is a secreted protease that is overexpressed in more than 70% of breast cancers. However, PAPP-A is a collagen-dependent oncogene. We initiated this study to test the effect of PAPP-A on the predisposition of post-partum breasts.

**Methods:**

We used PAPP-A mouse models for the analysis of its effect on virgin, involuting, or post-partum mammary glands. We performed second-harmonic generation microscopy for the analysis of collagen, defined tumor-associated collagen signature (TACS), the rate of mammary tumors, and the status of the collagen-DDR2-Snail axis of metastasis. We knockdown DDR2 by CRISPR and performed invasion assays. A transcriptomic approach was used to define a PAPP-A and parity-dependent genetic signature and assess its correlation with breast cancer recurrence in humans.

**Results:**

We confirmed that post-partum mammary glands have a higher level of collagen than virgin glands and that this collagen is characterized by an anti-proliferative architecture. However, PAPP-A converts the anti-proliferative post-partum collagen into pro-tumorigenic collagen. We show that PAPP-A activates the collagen receptor DDR2 and metastasis. Further, deletion of DDR2 by CRISPR abolished the effect of PAPP-A on invasion. We defined a PAPP-A-driven genetic signature that identifies patients at higher risk of metastasis.

**Conclusions:**

These results support the notion that information about pregnancy may be critical in the prognosis of breast cancer as passage through a single pregnancy predisposes to the oncogenic action of PAPP-A. Our data indicate that history of pregnancy combined with the expression of PAPP-A-driven genetic signature may be useful to identify patients at higher risk of metastatic disease.

**Electronic supplementary material:**

The online version of this article (10.1186/s13058-019-1142-z) contains supplementary material, which is available to authorized users.

## Background

Diverse factors contribute to the etiology of breast cancer, including parity and breastfeeding [1–5]. Pregnancy however has a dual effect on breast cancer risk. On one hand, at a young age (before the age of 25), pregnancy confers a lifelong protective effect [1]. On the other hand, pregnancy is associated with a transient risk of developing breast cancer in all women, and this risk peaks 6 years after birth [1–4]. In fact, a recent meta-analysis combining the results of 15 studies revealed that women remain at higher risk of breast cancer following pregnancy for more than 20 years, suggesting that healthcare providers should consider parity as a risk factor [6]. Furthermore, the relative risk directly correlates with the age of the mother. Notably, women above the age of 35 at the time of their first pregnancy remain at higher risk of developing breast cancer compared to nulliparous women for over three decades after pregnancy [1, 2]. Therefore, contrary to the current definition of pregnancy-associated breast cancer (PABC), which is empirically restricted to breast cancers diagnosed within the first 2 years post-partum, the diagnosis of PABC vs. non-PABC is very challenging [1–4].

As women in developed countries are steadily delaying childbearing to a later age, PABC incidence is expected to drastically increase, yet the link between pregnancy and breast cancer remains a poorly understood area of breast cancer research [3, 4, 7]. This is particularly significant as PABC are classically more aggressive than non-PABC. PABC patients typically exhibit higher rates of recurrence, metastasis, and poorer survival rates compared to age-, stage-, and hormone-matched nulliparous controls [1, 8, 9]. These observations highlight the need for reliable biomarkers and a deeper understanding of the molecular underpinnings of PABC.

Recent breakthroughs have identified involution as a pro-tumorigenic phase of breast development [8–10]. Involution is a transient event that is initiated by the cessation of lactation and which resets the mammary tissue architecture to a non-pregnant, post-partum state. Involution involves a drastic remodeling of the breast through a complex coordination of selected epithelial cell apoptosis, extracellular matrix remodeling, and adipogenesis. The signaling pathways involved share similar characteristics to wound healing and tumor-promoting microenvironments [10, 11].

One key feature contributing to the pro-tumorigenic effect of involution is the increased deposition of collagen [8, 9, 12]. High collagen abundance and stromal stiffening can disrupt the homeostasis of the extracellular matrix and are associated with an increased risk for breast cancer, cell invasion, and metastasis [13–20]. Importantly, the collagen of an involuting breast transiently adopts an altered architecture, referred to as tumor-associated collagen signature 3 (TACS3) [8, 13]. In contrast to normal collagen, TACS3 is characterized as fibrillar, linearized collagen fibers perpendicular to the tumor or ductal border [8, 13]. TACS can be further classified as TACS1, 2, or 3 depending on the angle of the collagen fiber [13]. TACS3 is associated with worst prognosis and most advanced stage of breast cancer [13, 17].

A post-partum breast, defined as a breast after the completion of involution and therefore does not have any of the wound healing characteristics of the involuting breast, retains the high collagen content observed in involution and is therefore different from breasts that have never gone through pregnancy [8, 14]. Unlike involution-collagen, however, post-partum collagen adopts a TACS1 architecture. Unlike TACS3, TACS1 collagen is anti-proliferative and has been postulated to contribute to the protective effect of pregnancy [13]. These observations raise the possibility that oncogenic events able to convert the post-partum collagen into an involution-like collagen may predispose post-partum breasts to the development of breast cancer.

Pregnancy-associated plasma protein A (PAPP-A) or pappalysin is a secreted protease that is widely overexpressed in breast cancer, and therefore, we seek to define its role in breast cancer [21]. We reported that pappalysin-1 (PAPP-A) transgenic mice develop PABC and characterized the effect of PAPP-A during involution [22]. The only known substrates of PAPP-A are IGFBP-4 and IGFBP-5, two negative regulators of IGF signaling [23–25]. Importantly, IGFBP-5 is a key regulator of involution [11]. Further, small nucleotide polymorphisms leading to a reduction in IGFBP-5 expression have been identified by genome-wide association studies (GWAS) as a risk factor of developing breast cancer [26]. However, the role of IGFBP-5 in breast cancer has largely escaped attention. Importantly, we found that collagen drastically enhances the proteolytic activity of PAPP-A and its ability to cleave IGFBP-5 [22]. This result suggests that the high collagen environment of involution is an absolute requirement to unleash PAPP-A oncogenic potential [22]. As post-partum breast is also characterized by elevated collagen, this observation raised the possibility that PAPP-A may contribute to the extended increased risk of breast cancer that persists for decades following pregnancy [1–4, 7]. Further, given that PABC have a higher rate of metastasis [4], we also aimed at testing whether PAPP-A plays a role in metastasis. Our results indicate that a single pregnancy is sufficient to predispose a breast to the oncogenic effect of PAPP-A and that the action of PAPP-A is not limited to the cleavage of IGFBP-5 but extend to the activation of a DDR2/Snail axis of metastasis. These new findings led to the identification of a genetic signature that identifies patients at higher risk of metastasis and is characterized by a profound remodeling of the extracellular matrix.

## Methods

### Animal procedures

Mice overexpressing PAPP-A in the mammary gland (MMTV-PAPP-A) in FVB/n background were generated as previously described (Takabatake et al). The animals were sacrificed, and the tissues were stored at − 80 °C for biochemical analyses. For the post-partum time course experiments, 2-month-old nulliparous FVB/n non-transgenic and PAPP-A transgenic females (*n* = 20 mice per type, 5 mice per group) were bred and subsequently housed separately post-fertilization. All females were kept with their litter for either a 2-day- or 2-week-long lactation period. Involution mice were sacrificed 12 days post-weaning, and late post-partum mice were sacrificed 2 months post-weaning. The mammary glands were harvested for histological analyses or as RNA and protein samples at − 80 °C.

### Histology and immunohistochemistry

Histological slides were prepared as 4 μm formalin-fixed sections embedded in paraffin and stained with H&E by the Oncological Sciences Department Histology core facility at Icahn School of Medicine at Mount Sinai. For immunostaining of paraffin-embedded sections, the samples were deparaffinized in xylene and rehydrated in water. Antigen retrieval was performed using citrate buffer pH 6 for 30 min at 95 °C. Peroxidase blocking was completed using endogenous peroxidase solution (Dako Dual Endogenous Enzyme Block #S20003) at room temperature for 15 min. Antibody Diluent (MP Biomedicals Normal Antibody Diluent #980631) was used for serum blocking for 30 min at room temperature. All buffer washes between incubations were completed with TBS. Primary antibody (1:100 Snail, Cell Signaling Technology L70G2 #3895) incubation was performed overnight at 4 °C in an enclosed moist chamber. Biotinylated mouse antibody (Jackson ImmunoResearch #115-065-205) diluted 1:200 in TBS for 1 h at room temperature was used for the secondary antibody incubation. Washes between antibody incubations were done with TBS with .04% Tween. Streptavidin peroxidase (Vector Labs #SA-5004) was incubated for 30 min at room temperature, followed by a TBS with .04% Tween and TBS wash. The slides were developed using AEC (Vector Labs #SK-4200) for 15 min at room temperature and mounted using VectaMount (Vector Labs #H-5501). Images were acquired using a Zeiss AX10 light microscope and scored according to the parameters shown.

### Second-harmonic generation microscopy

All mouse mammary gland second-harmonic generation (SHG) images were captured on an Olympus FV1000MPE Fluoview multiphoton microscope (Tokyo, Japan) in the Microscopy CoRE at the Icahn School of Medicine at Mount Sinai, supported with funding from NIH Shared Instrumentation Grant (1S10RR026639-01). Image acquisition was done using an Olympus XLPlanN 25×/1.05 numerical aperture water immersion lens with the excitation wavelength set at 900 nm. Imaging specs included Coherent Chameleon Vision II Ti:S laser (Santa Clara, CA) with a 680- to 1080-nm tuning range, dispersion compensation, and 140 fs pulse width. Signal collection was performed by backward imaging with a 420- to 460-nm band-pass filter, a 485 dichroic mirror (GR/XR filter cube, Olympus), and an external detector. Images were acquired at a 512 × 512 pixel resolution with consistent power levels. Protocol for collagen intensity vs. distance analysis was adapted from Lyons et al. and performed in six replicate ducts per mouse in five mice per group (*n* = 30 ducts per group). SHG images were opened in ImageJ (version 1.46r), and a rectangular selection of 50 × 100 pixels was made bordering the ductal edge in four compass directions. Each region of interest (ROI) was analyzed using the profile plot function, and intensity of the SHG signal over a 40-μM distance was averaged as the signal for a single duct.

### Tumor-associated collagen signature analysis

SHG images of the mammary ducts and tumors (acquired as described in the “Second-harmonic generation microscopy” section) were imported into the curvelet-based alignment analysis software CurveAlign (version 2.2 R2012a), available through the Laboratory of Optical and Computational Instrumentation at the University of Wisconsin-Madison. The borders of the mammary ducts and tumors were manually indicated, and the CurveAlign software measured the angle and quantity of collagen fibers with respect to the designated borders within a preset pixel distance. The individual curvelet angles were reported as an output value between 0 and 90. Based on prior literature, angles between 60 and 90 were characterized as a positive TACS3 region, angles between 30 and 60 as TACS2, and angles 0–30 as TACS1. The number of individual collagen fibers having an angle of interaction within each of the three signatures was counted and normalized to the total number of collagen fibers assessed within an ROI by the software.

### Masson’s trichrome collagen stain and analysis

The sections were deparaffinized in xylene and rehydrated in water. Masson’s trichrome staining kit (DAKO, Cat#AR173) was performed following the manufacturer’s procedure protocol and mounted using Permount toluene solution (Thermo Fisher Scientific). Triplicate images of the stained sections were collected for representative areas of 2560 × 1920 pixels (0.44 μm/pxls) using a Zeiss AX10 light microscope. The positive blue stain was converted to black using Adobe Photoshop CS5’s (version 12.0 × 64) magic wand tool. The images were imported into ImageJ (version 1.46r), and the black selections were quantified as percent area positive for collagen. The images were normalized against the periphery of the duct epithelia in which the final ratio represents collagen/epithelia.

### Cell culture and treatment conditions

MCF-7^PAPP-A^ and MCF-7^PAPP-AE483Q^ cells were generated as previously described (Takabatake et al). MCF-7, MCF-7^PAPP-A^, MCF-7^PAPP-A/DDR2 KO^, and MCF-7^PAPP-AE483Q^ cells were grown in Dulbecco’s modified Eagle’s medium (DMEM) supplemented with 10% fetal bovine serum and 1% penicillin/streptomycin at 37 °C/5% CO_2_. rIGF-1 (Preprotech, #100-11) treatments were done in serum-free media at 10 nM for indicated time points. Imatinib (R&D Systems, #59-061-00) and PQ401 (Enzo Life Sciences ALX-270-459-M005) treatments were performed at indicated concentrations in complete media for 48 h, and untreated samples were treated with vehicle control (DMSO) for 48 h. PAPP-A- and PAPP-A E483Q-conditioned media treatments were performed at 10 ng/mL in complete media for 24 h. Cells treated with conditioned media were seeded on collagen I-coated plates 24 h prior to treatment. Preparation of collagen-coated plates was performed as follows: type I rat tail collagen (Corning, #354236) was diluted at 50 μg/mL in 0.1 nM HCl and incubated on plates at room temperature for 1 h followed by two PBS washes.

### Media collection and PAPP-A ELISA

MCF-7^PAPP-A^ and MCF-7^PAPP-AE483Q^ cells were grown to 80% confluence in complete media. Media were then switched to serum-free and collected 24 h later, and PAPP-A protein was concentrated from media using 50 kDa cutoff Amicon Ultra-15 Centrifugal Filter Units (EMD Millipore, #UFC905024) spun at 5000*g* for 20 min at 4 °C. Concentrated condition media were then stored at − 20 °C, used for rIGFBP-5 protease assay, or quantified by ELISA. PAPP-A concentration was measured using Quantikine PAPP-A ELISA (R&D Systems, # DPPA00) following the manufacturer’s protocol adapted with a primary incubation for 18 h at 4 °C and PAPP-A conjugate incubation for 6 h at room temperature.

### IGFBP-5 protease assay

Cell-free protease assays were performed using PAPP-A protein secreted in 24 h serum-free culture media from MCF-7 PA and MCF-7 PA E483Q cells. Culture media from parental MCF-7 were used as a negative control for PAPP-A protein in cell media. Media were collected and concentrated as described above. Fifty nanograms of rIGFBP-5 (Abcam, #ab49835) was co-incubated with 15 μL of concentrated media and 15 μL of DMEM for 3 h at 37 °C. The reaction was inactivated by the addition of 1× Laemmli sample buffer to 40 μL final volume and boiling for 5 min. Degradation of IGFBP-5 in samples was then detected by western blot.

### Immunoblot

Cell lysates were prepared in RIPA lysis buffer (25 mM Tris HCl, pH 7.6, 150 mM NaCl, 1% NP-40, 0.1% SDS, 1% sodium deoxycholate) supplemented with protease and phosphatase inhibitors. Thirty micrograms total protein in 1× Laemmli buffer per sample was separated on a 10% SDS-glycine polyacrylamide gels ran at 80 V for 30 min and 200 V for 45 min. Proteins were transferred to nitrocellulose membranes (GE Healthcare) for 1.5 h at 85 V. Membranes were blocked in 5% milk in TBS-T and incubated on a rotator overnight at 4 °C in the following primary antibodies: DDR2 1:250 (Cell Signaling Technology #12133S), p-DDR2 Y740 1:600 (R&D Systems, #MAB25382), Snail 1:1000 (Cell Signaling Technology L70G2 #3895), Larp6 1:600 (Abnova, #H00055323-B01P), IGFBP-5 1:1000 (EMD Millipore, #06-110), PAPP-A 1:200 (Santa Cruz Biotechnology, # sc-50518), phospho-Akt (Ser473) 1:1000 (Cell Signaling Technology 587F11 #4051), Akt 1:10,000 (Cell Signaling Technology #9272), and actin 1:10,000 (EMB Millipore #MAB1501R). After three washes in TBS-T, and 10-min 10% milk/TBS-T incubation, the membranes were incubated in secondary anti-rabbit or anti-mouse antibodies conjugated to horseradish peroxidase (Thermo Fisher Scientific or KwikQuant) in 10% milk/TBS-T for 1 h at room temperature. Signal was detected using ECL (GE Healthcare, #RPN2106 or KwikQuant) following the manufacturer’s protocols. TBS-T was prepared with 0.1% Tween.

### RNA isolation and RT-qPCR

Total RNA was extracted from cell lines using TRIzol (Invitrogen) following the manufacturer’s protocol. One hundred nanograms of each triplicate sample was used in real-time RT-PCR using One-Step SYBR PrimeScript RT-PCR kit (Takara, Cat#RR086A) following the manufacturer’s protocols. Thermal cycle program for DDR2 primers: 50 °C 2 min, 95 °C 20 s, 40 cycles of 95 °C 3 s, 60 °C 30 s, and 72 °C 5 s, and Col1a1 primers: 42 °C 5 min, 94 °C 5 min, 30 cycles of 94 °C 12 s, 60 °C 8 s, 72 °C 8 s, both followed by a melt curve of 65 °C to 95 °C in 0.4 °C increments. Experiments were carried out in triplicate for each data point, and relative expression was determined using 2^−ΔΔCτ^ method.

**Table:** 

Gene	Forward	Reverse
Human DDR2	5′-TCA CCC AGA CCC ATG AAT AC-3′	5′-GGG AAG GAA ATG GCA TTA GG-3′
Human Col1a1	5′-TCT GCG ACA ACG GCA AGG TG-3′	5′-GAC GCC GGT GGT TTC TTG GT-3′
Human β-actin	5′-ATC CTC ACC CTG AAG TAC CC-3′	5′-TAG AAG GTG TGG TGC CAG AT-3′

### Transwell in vitro invasion assay

Cell permeable membrane inserts (Falcon, #08-771-21) were coated with 200 μL of diluted 1:100 growth factor-reduced Matrigel basement membrane matrix (Corning, #356230) in cold PBS. Inserts were placed in 24-well plates (Falcon), and artificial extracellular matrix was allowed to polymerize for 2 h at room temperature. Following polymerization incubation, excess Matrigel solution was removed by pipette. Fifty thousand cells in 500 μL of serum-free DMEM were seeded atop the polymerized Matrigel in the inserts. Five hundred microliters of complete DMEM containing 10% FBS was added to each lower chamber. Invasion assays were incubated at 37 °C/5% CO_2_. After 48 h, invaded cells were fixed and stained using the Hema 3 Manual Staining Stat Pack (Thermo Fisher Scientific, #23-123-869) according to the manufacturer’s protocol and mounted using Permount toluene solution (Thermo Fisher Scientific). All experiments were done in technical triplicates.

### Xenografts using MCF-7, MCF-7^PAPP-A^, and MCF-7^PAPP-A DDR2 KO^ cells

A week prior to cell injection, a 60-day release 0.5 mg β-estradiol 17-acetate pellet (Innovative Research of America, #SE-271) was inserted in the cervical region of virgin female athymic nude mice aged 2 months. Using a 25-G needle, 2.5 million cells (MCF-7 parental or MCF-) in 100 μL of Matrigel basement membrane matrix (Corning, #354234) were injected in the left inguinal mammary fat pads and 2.5 million cells in 100 μL of 1:1 Matrigel/type 1 collagen mixture (collagen: Corning, #354236) were injected in the right inguinal mammary fat pads (*n* = 5 mice, 10 tumors total). Palpable tumors were measured by caliper 3 times per week for 3 weeks beginning 14 days post-injection. Final tumor volumes were recorded by weight, and tumors were harvested for histological analyses or as RNA and protein samples at − 80 °C. The lungs were collected for histological analysis following perfusions with sterile PBS and 10% formalin.

### MTT assay

MCF-7 and MCF-7^PAPP-A^ cells were seeded at 50% confluence and treated with imatinib and PQ401 at the indicated time points in triplicate samples for 48 h. Following treatment, complete media with MTT at 0.5 mg/mL was added to each well and incubated at 37 °C for 3 h protected from light. After incubation, and DMSO was used as MTT solvent and each will be mixed thoroughly to homogenize the color and subsequently read at absorbance OD = 570 nm. The samples were averaged and normalized to the vehicle control sample.

### Met1 xenograft and conditioned media injections

Two-month-old virgin female FvBN mice were bred and then housed separately post-fertilization. Pregnant mice were checked daily for litter drop and then underwent a lactation period of 2 days. Mice were injected with met1 cells at a time point of post-partum 2 months post-lactation. Met1 cells were kindly provided by Dr. Emily Gallagher at Mount Sinai. Two hundred fifty thousand met1 cells in 100 μL of control MCF-7- or PAPP-A-conditioned media at 0.1 ng/μL were injected into the upper right and left inguinal mammary fat pads (*n* = 5 mice, 6 tumors per group). The lower left and right inguinal mammary fat pads received injections of MCF-7-concentrated media or concentrated PAPP-A from MCF-7^PAPP-A^ media at 0.1 ng/μL alone (*n* = 5 mice, 6 mammary glands per group, PAPP-A concentrated as described above). Subsequently, every 2 days for 3 weeks following the initial injection, 100 μL of control media or 0.1 ng/μL PAPP-A containing media was injected into the upper and lower left and right inguinal mammary fat pads or Met1 xenografts. All injections were done using a 25-G needle. Final tumor volume was recorded by weight, and all tumors and mammary glands were harvested for histological analysis. The lungs were collected for histological analysis following perfusions with sterile PBS and 10% formalin. Clusters of cancer cells were detected by H&E and counted as micrometastases.

### Gene set analysis

Publicly available primary breast cancer gene expression and phenotype data published by Kao et al. (GSE20685) was downloaded from Gene Expression Omnibus [27]. *PAPP-A/SNAI1/COL1A1* gene signature was composed of the probes for each gene with the highest average expression across all samples. The probes used for each gene are as follows: *PAPP-A* (201981_at), *SNAI1* (219480_at), and *COL1A1* (202310_s_at)*.* The secondary validation dataset in Additional file 4: Figure S4 was collected from Chanrion et al. (GSE9893), and the probes used were *PAPP-A*: H200006224, *COL1A1*: H200008097, and *SNAI1*: H200004861 [28]. Normalized expressions of *PAPP-A/SNAI1/COL1A1* were averaged to calculate an expression score for each patient (*n* = 327). The top 50% of patients with the highest expression scores were designated as *PAPP-A/SNAI1/COL1A1* high (*n* = 163), and the bottom 50% were designated as *PAPP-A/SNAI1/COL1A1* low (*n* = 164). Differential gene expression between the two groups of patients was calculated using by LIMMA [29]. Genes with a false discovery rate (FDR) less than 0.05 were ranked by Log_2_ fold change and used for pre-ranked gene set enrichment analysis (GSEA) using GenePattern [30]. Enrichment scores were determined as a running sum statistic at maximum deviation from zero.

### Survival analysis

For the outcome analysis, we constructed Kaplan-Meier curves for time to distant metastasis as the endpoint. This was defined as the time from primary disease diagnosis to the detection of distant metastasis. Differences between patients with a high and low score of our predefined *PAPP-A/SNAI1/COL1A1* signature were evaluated using the log-rank test. Patients who died without metastasis were censored at the time of death. The analysis was performed using R Packages Survival, Survminer, and RMS [31].

### CRISPR-Cas9 genome editing

For lentiviral vector production, HEK293T cells were used in standard cell culture conditions with DMEM supplemented with 10% FBS. Cells at 80% confluence were co-transfected using Lipofectamine 2000 (Invitrogen #11668-019) following the manufacturer’s protocol with 12 μg of lentiviral vector, 9 μg of psPAX2, and 3 μg of pMD2.G. Fresh media (DMEM supplemented with 10% FBS and penicillin/streptomycin) were exchanged 16 h post-transfection, and supernatants were collected 48 h post-transfection and sterile filtered (.45 μm). Target cells (MCF-7 and MCF-7^PAPP-A^) were infected with the lentiCRISPRv2 (Addgene #52961) plasmid lentiviral supernatant described above and polybrene (8 μg/mL) for 24 h. Cells were selected with puromycin for 2 weeks prior to clonal isolation and expansion. gRNAs (see oligo sequence below) targeting DDR2 were designed using the publicly available MIT CRISPR Design program.

**Table:** 

DDR2 gRNA 1	5′-CAC CGT TCA TGG AGT GGT CGG TGA C-3′
DDR2 gRNA 2	5′-AAA CGT CAC CGA CCA CTC CAT GAA CGG TGC-3′

## Results

### PAPP-A transgenic mice sustain involution-like collagen in post-partum mammary glands in vivo

To test whether the expression of PAPP-A affects the amount, distribution, or orientation of collagen in post-partum mammary glands, we harvested the mammary glands from parous PAPP-A transgenic or non-transgenic mice during involution (12 days post-weaning) or post-partum (2 months post-weaning). We previously shown that due to the use of a truncated MMTV promoter, where the hormonal responsive element is deleted, the expression of PAPP-A in our mouse model is constitutive but is not enhanced by pregnancy [22]. We showed that PAPP-A levels are similar in virgin, pregnant, lactating, involuting, and post-partum mammary glands [22].

**Fig. 1:**
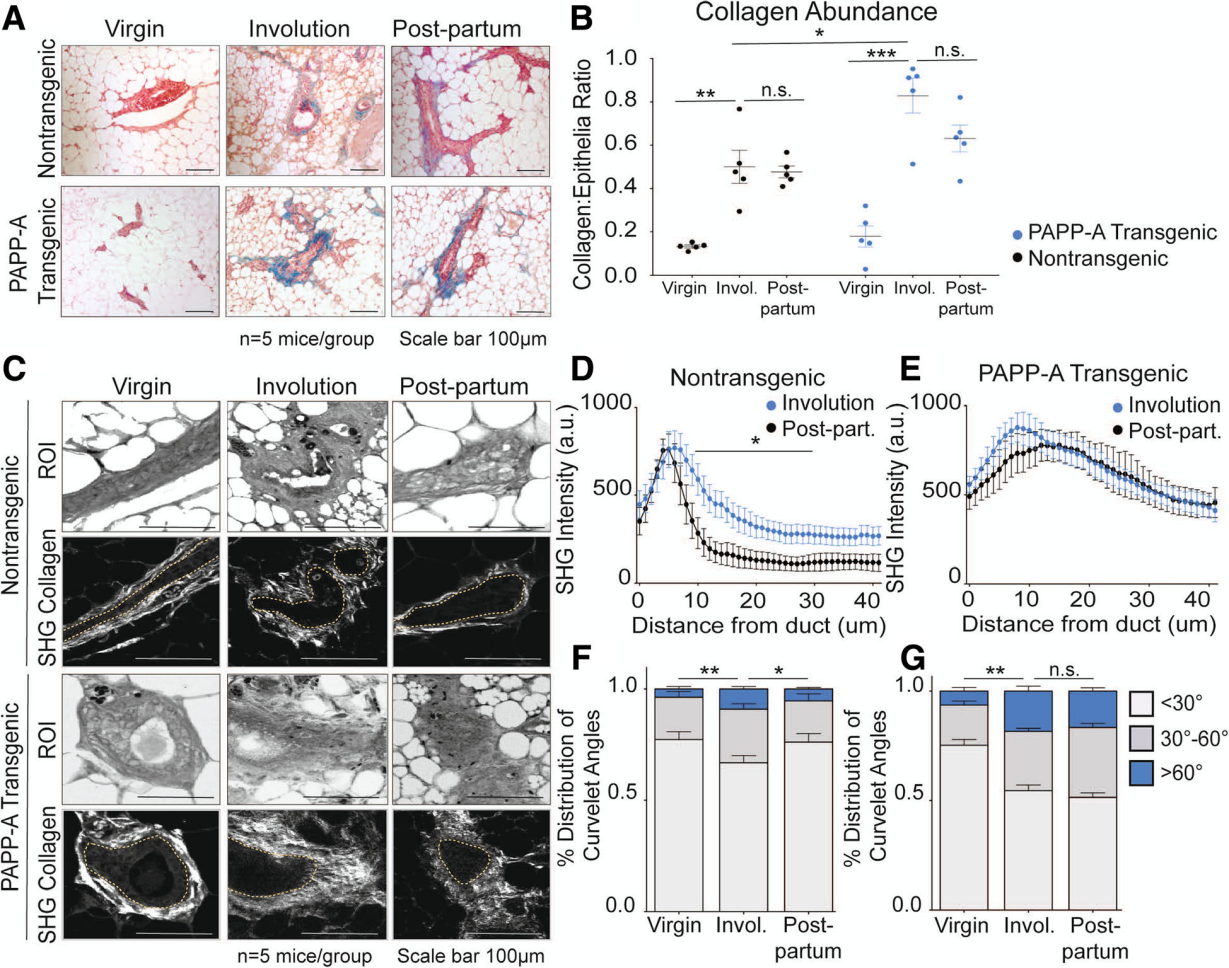
PAPP-A sustains an altered collagen phenotype in post-partum mammary glands. **a** Representative images of Masson’s trichrome collagen stain (blue) on virgin, involuting, or late post-partum mammary glands from non-transgenic and PAPP-A transgenic mice. *n* = 5 mice per group. Scale bar 100 μm. **b** Quantification of collagen per epithelial region by Masson’s trichrome stain on non-transgenic and PAPP-A transgenic virgin, involuting, or late post-partum mammary glands from **a**. *n* = 5 mice per group, each point represents the average of ten ducts per mouse per time point. Mean ± SEM, unpaired *t* test with Welch’s correction: **p < 0.005, ***p < 0.0005. **c** Representative second-harmonic generation (SHG) imaging of collagen on magnified ducts of histological sections of non-transgenic or PAPP-A transgenic virgin, involuting, or late post-partum mammary glands (lower panels). The yellow dotted lines indicate mammary ducts borders; collagen is seen in white. Corresponding regions of interest (ROI) are shown in top panels. *n* = 5 mice per group. Scale bar 100 μm. **d** Graph of collagen intensity relative to the distance from the mammary duct borders of SHG images of non-transgenic mammary glands in **c**. Mean ± SEM. *n* = 5 mice per group, unpaired *t* test with Welch’s correction: *p < 0.05. **e** Graph of collagen intensity relative to the distance from the mammary duct borders of SHG images of PAPP-A transgenic mammary glands in **c**. Mean ± SEM. *n* = 5 mice per group. **f** Quantification of TACS3 per total curvelets analyzed in virgin, involuting, or late post-partum mammary glands of non-transgenic mice using the CurveAlign software. TACS3 characterized as curvelet angles 60–90 relative to the ductal border. *n* = 5 mice per group. Mean + SEM, unpaired *t* test with Welch’s correction (comparisons between the TACS3 group only). *p < .05, **p < 0.005. **g** Quantification of TACS3 per total curvelets analyzed in virgin, involuting, or late post-partum mammary glands of PAPP-A transgenic mice using the CurveAlign software. TACS3 characterized as curvelet angles 60–90 relative to ductal border. *n* = 5 mice per group. Mean + SEM, unpaired *t* test with Welch’s correction (comparisons between the TACS3 group only). **p < 0.005

First, the amount of collagen in each group was analyzed using Masson’s trichrome staining. As internal controls, we confirmed that the amount of collagen in the non-transgenic females is increased during involution (Fig. 1a, b) and is maintained in the post-partum mammary glands (Fig. 1a, b) [8]. Further, in the PAPP-A transgenic mice, we confirmed that while the level of collagen in virgin females is not significantly different from that in non-transgenic virgin females, collagen during involution is significantly higher in PAPP-A transgenic mice compared to non-transgenic mice (Fig. 1a, b) [22]. We then analyzed the level of collagen in the post-partum glands in the PAPP-A transgenic mice. We found that the level of collagen remains higher in the post-partum mammary glands in PAPP-A transgenic mice (Fig. 1a, b).

Second, we then performed a second-harmonic generation to determine the distribution and orientation of collagen (Fig. 1c). Again as internal controls, we confirmed that in the non-transgenic mice, the collagen during involution shows a wide distribution as indicated by the presence of collagen across longer distances from the ducts and that in contrast, collagen is closer to the mammary duct borders, which is a characteristic of TACS1, in the post-partum mammary glands (Fig. 1d) [8]. We also confirmed that in the PAPP-A transgenic mice, collagen spreads over a wider range of distances from the ducts during involution compared to the non-transgenic mice (Fig. 1e) [22]. We then analyzed the distribution of collagen in the post-partum glands in the PAPP-A transgenic mice. We found that in sharp contrast to the non-transgenic mice, the same wide distribution was maintained in the post-partum glands in transgenic mice (Fig. 1e).

Third, we scored the angle of collagen fiber orientation. TACS3 regions are quantified as percent number of individual collagen fibers (curvelets) at an angle of 60–90° relative to the ductal border [13]. The total curvelets were analyzed using the CurveAlign software, and regions of interest showing an angle of collagen at more than 60° relative to the duct border were scored as TACS3. We found that in the non-transgenic mice, the levels of TACS3 increases during involution but are decreased in the post-partum glands (Fig. 1f). In contrast, in the PAPP-A transgenic mice, TACS3 collagen was increased during involution and was maintained at similar levels in the post-partum glands (Fig. 1g).

We concluded that the constitutive expression of PAPP-A in the mammary gland maintains the elevation in the amount, distribution, and orientation of collagen in the post-partum mammary gland. These alterations result in the persistence of involution-like collagen into the post-partum mammary glands. Considering that the collagen of involution is pro-tumorigenic, this result raises the possibility that the expression of PAPP-A, after involution is completed, in a post-partum mammary gland may contribute to the development of PABC.

### Injections of PAPP-A in post-partum mammary glands of non-transgenic mice convert post-partum collagen into involution-like collagen and promote PABC

In contrast to the PAPP-A transgenic mice, in human breast cancers, rather than being constitutive, the expression of PAPP-A is sporadic and can occur at any time before or after pregnancy. Notably, we show that PAPP-A is a transcriptional target of mutant p53 suggesting that mutations in p53 represent one mechanism by which PAPP-A is abnormally overexpressed in breast cancer [32–34]. Having established that constitutive expression of PAPP-A in the PAPP-A transgenic mice maintains an involution-like collagen in the post-partum glands (Fig. 1), we next investigated whether sporadic expression of PAPP-A in an established post-partum mammary gland of non-transgenic mice is able to convert the post-partum collagen architecture into an involution-like collagen architecture. To test this possibility, we established a cohort of non-transgenic post-partum mice (2 months post-weaning). In parallel, we collected media from MCF-7 cells overexpressing PAPP-A (MCF-7^PAPP-A^), which we previously characterized and shown to secrete proteolytically active PAPP-A [22]. We then concentrated the PAPP-A from the media and injected PAPP-A (PAPP-A media) or control media concentrated from MCF-7 cells into the post-partum mammary glands of the non-transgenic mice every 2 days for 3 weeks.

**Fig. 2:**
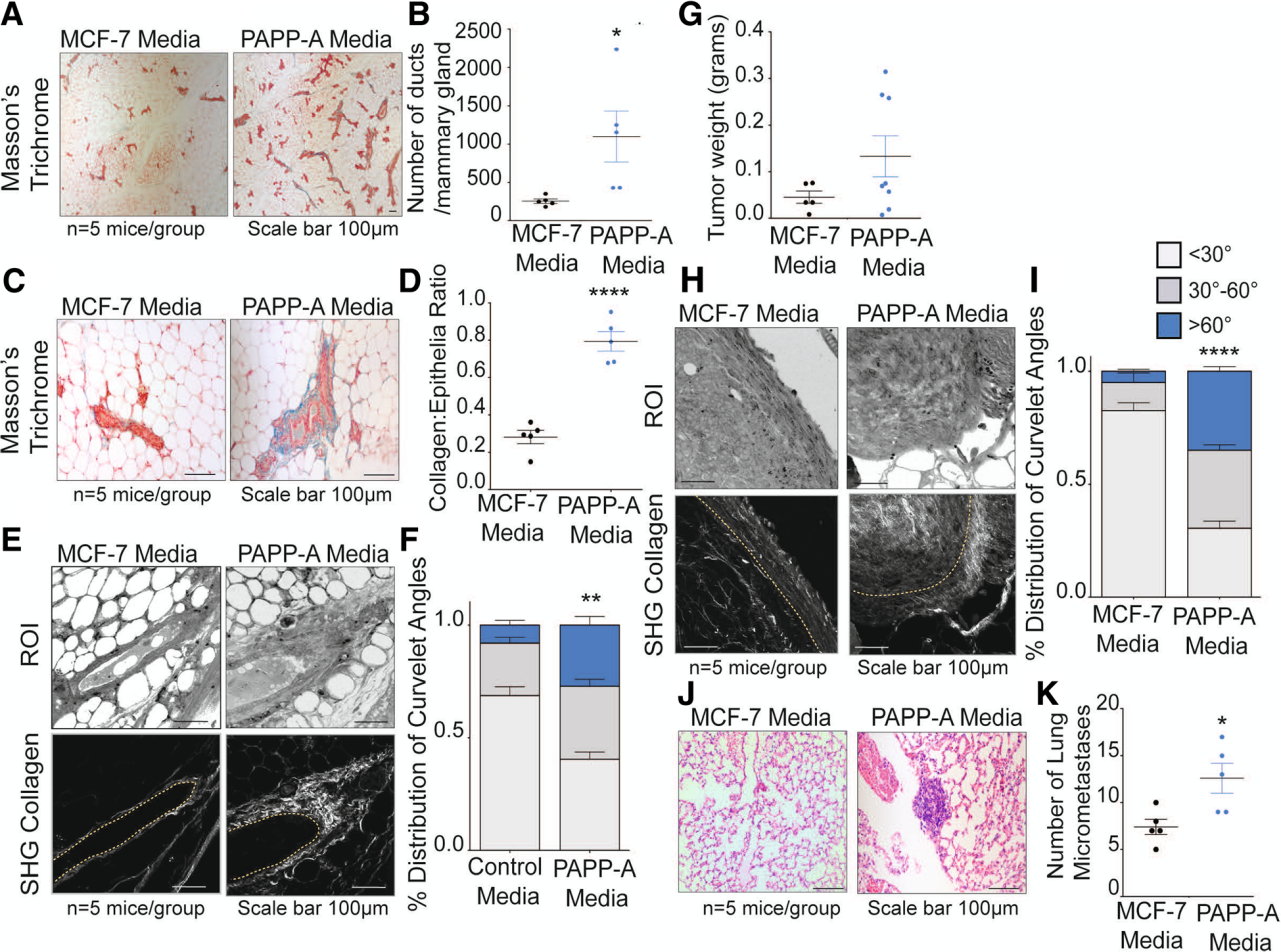
PAPP-A converts post-partum collagen into an altered, pro-tumorigeneic involution-like collagen phenotype. **a** Representative images of the mammary ducts stained with Masson’s trichrome collagen (blue) on non-transgenic late post-partum mammary glands treated with control or PAPP-A injections. *n* = 5 mice per group. Scale bar 100 μm (images were taken at × 5). **b** Quantification of the number of ducts per mammary gland from non-transgenic post-partum mice treated with control or PAPP-A media injections. *n* = 5 mice per group, each point represents the average of each mouse per group. Mean ± SEM, unpaired *t* test: *p < 0.05). **c** Representative images of Masson’s trichrome collagen stain (blue) on non-transgenic post-partum mammary glands treated with control or PAPP-A media injections. *n* = 5 mice per group. Scale bar 100 μm. **d** Quantification of collagen per epithelial region by Masson’s trichrome stain on non-transgenic post-partum mammary glands treated with control or PAPP-A media injections. *n* = 5 mice per group, each point represents the average of ten ducts per mouse per group. Mean ± SEM, unpaired *t* test with Welch’s correction. ****p < 0.0001. **e** Representative second-harmonic generation (SHG) imaging of collagen on magnified ducts of histological sections of non-transgenic late post-partum mammary glands treated with control or PAPP-A media injections (lower panels). Yellow dotted lines indicate the mammary duct borders. Corresponding regions of interest (ROI) are shown in the top panels. *n* = 5 mice per group. Scale bar 100 μm. **f** Quantification of TACS3 per total curvelets analyzed in non-transgenic post-partum mammary glands treated with control or PAPP-A media injections using the CurveAlign software. TACS3 characterized as curvelet angles 60–90 relative to the ductal border. *n* = 5 mice per group. Mean + SEM, unpaired *t* test with Welch’s correction (comparisons between the TACS3 group only). **p < 0.005. **g** Tumor weights at time of harvesting Met1 xenograft tumors from non-transgenic post-partum mice treated with control or PAPP-A media injections. *n* = 5 mice per group, two xenografts injected per mouse. In the MCF-7 media group, 5/10 tumors took while 8/10 tumors grew in the PAPP-A media group. Each point represents a tumor weight. Mean ± SEM. **h** Representative second-harmonic generation (SHG) imaging of collagen on magnified histological sections of Met1 xenograft tumors in non-transgenic late post-partum mice treated with control or PAPP-A media injections (lower panels). Yellow dotted lines indicate the mammary duct borders. Corresponding regions of interest (ROI) are shown in the top panels. *n* = 5 mice per group. Scale bar 100 μm. **i** Quantification of TACS3 per total curvelets analyzed in Met1 xenograft tumors in non-transgenic late post-partum mice treated with control or PAPP-A media injections using the CurveAlign software. TACS3 characterized as curvelet angles 60–90 relative to the ductal border. *n* = 5 mice per group. Mean + SEM, unpaired *t* test with Welch’s correction (comparisons between the TACS3 group only). ****p < 0.0001. **j** Representative images of lung micrometastases in post-partum non-transgenic mice xenografted with Met1 cells and treated with control or PAPP-A media injections. *n* = 5 mice per group. Scale bar 100 μm. **k** Quantification of lung micrometastases in post-partum non-transgenic mice xenografted with Met1 cells and treated with control or PAPP-A media injections. *n* = 5 mice per group, each point represents the average of lung micrometastases per mouse. Mean ± SEM, unpaired *t* test with Welch’s correction: *p < 0.05

We found that injections of PAPP-A in the post-partum mammary glands of non-transgenic mice induce a remarkable increase in proliferation, indicated by the significantly higher number of ducts per mammary glands (Fig. 2a, b). Further, the post-partum mammary glands injected with PAPP-A had significantly higher collagen abundance (Fig. 2c, d). SHG imaging of collagen orientation and CurveAlign analysis revealed that post-partum mice injected with PAPP-A adopted a TACS3 phenotype, compared to the mammary glands injected with control media, which exhibited predominantly TACS1 collagen (Fig. 2e, f). As a control, we also injected PAPP-A media into the mammary glands of virgin non-transgenic mice. However, injections of PAPP-A had no significant effect on the proliferation or collagen in the mammary glands of virgin mice (Additional file 1: Figure S1A, B). These results suggest that the sporadic presence of PAPP-A is sufficient to convert post-partum collagen into a high TACS3 involution-like collagen architecture.

Considering that involution-collagen is pro-tumorigenic, we then tested whether the effect of sporadic expression of PAPP-A affects tumor growth. To address this possibility, we used Met1 breast epithelial cancer cells, however, as these cells are known to form aggressive metastatic tumors in mice, we injected only 250,000 cells in order to increase the probability to detect differences between the groups. Met1 cells were injected into the post-partum mammary glands of non-transgenic mice along with control media or media containing PAPP-A followed by injections of the respective media every 2 days for 3 weeks. We found that tumors injected with PAPP-A are on average larger compared to the control group, but this difference did not reach statistical significance (Fig. 2g). In addition, CurveAlign analysis of the collagen orientation on these tumors revealed that the mammary tumors injected with PAPP-A had a significantly higher frequency of TACS3 regions compared to the control group (Fig. 2h, i).

As TACS3 is associated with increased metastasis [13], we analyzed these mice for lung metastases. Consistent with the higher TACS3 phenotype, mice injected with PAPP-A had a substantial increase in the frequency of micro-metastases to the lungs compared to the control group within this short period of time (Fig. 2j, k).

Taken together, these results indicate that sporadic expression of PAPP-A between pregnancies is able to convert the elevated levels of collagen of a post-partum gland to provide a pro-tumorigenic microenvironment. These results therefore imply that the abnormal gain of the overexpression of PAPP-A at any time after a first pregnancy may be sufficient to promote the development of PABC. Further, they indicate that PAPP-A also enhances the metastatic potential of cancer cells.

### PAPP-A promotes a DDR2/Snail signaling axis of invasion through collagen mRNA stabilization and IGF signaling in vitro

In addition to a structural role of collagen in metastasis [13, 35], collagen activates the discoidin domain receptors DDR1 and DDR2 [36–38]. DDR1 and 2 are frequently overexpressed in breast cancers and have recently been implicated in breast cancer metastasis [36, 39–41]. DDR2 has been linked with the invasive TACS3 phenotype, while the loss of DDR2 is associated with a non-invasive collagen signature, TACS2 [40, 41].

**Fig. 3:**
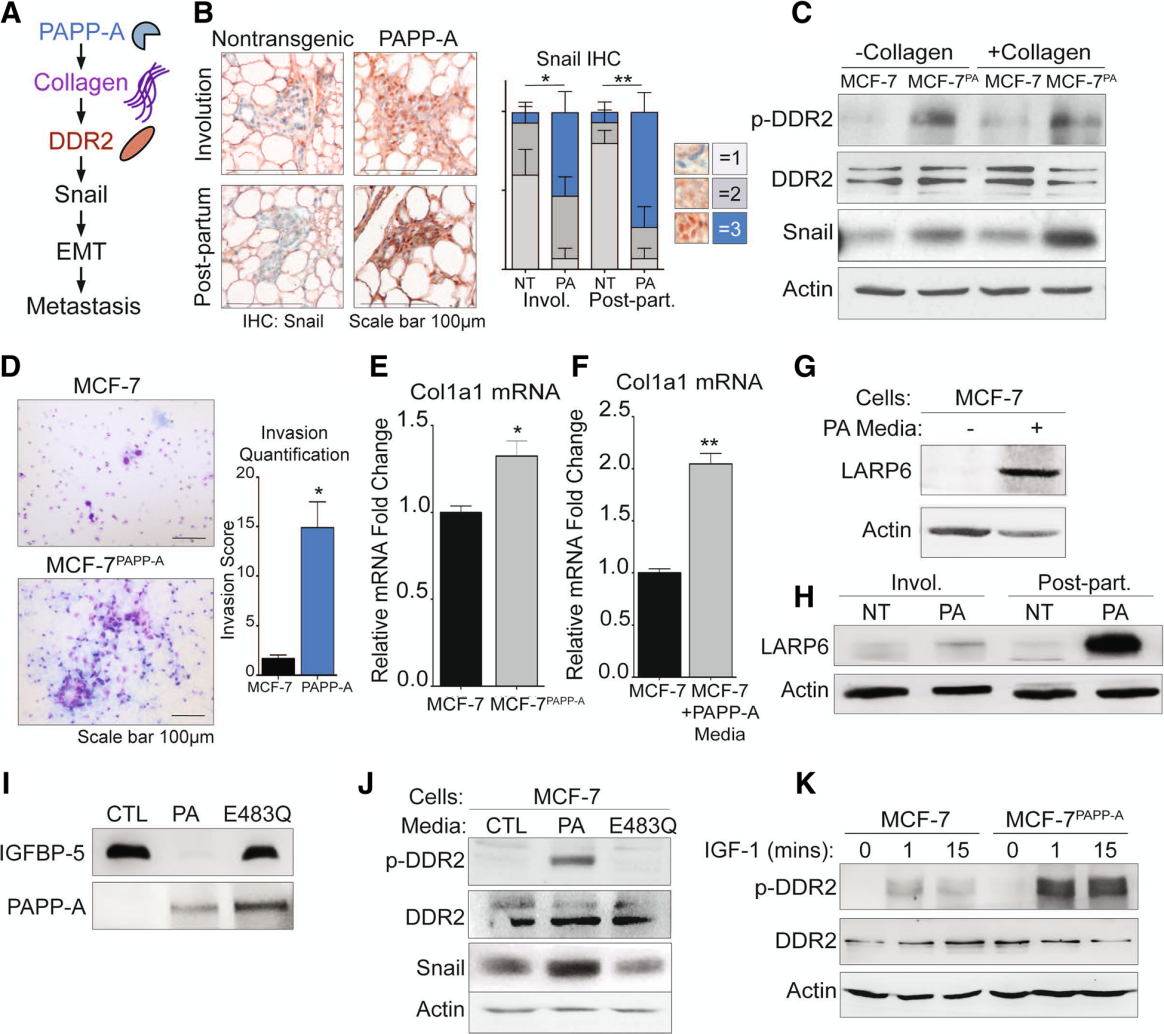
PAPP-A promotes DDR2 activation through collagen mRNA stabilization and IGF signaling in vitro. **a** Schematic of the proposed mechanism of activation of DDR2/Snail signaling axis of invasion [39]. **b** Representative images of Snail IHC (Snail: red; nuclei: blue) of virgin, involuting, or post-partum mammary glands from non-transgenic or PAPP-A transgenic mice. *n* = 5 mice per group. Quantification of Snail IHC is shown, *n* = 5 mice per group. Mean ± SEM, unpaired *t* test with Welch’s correction (comparisons between the score 3 group only). *p < 0.05, **p < 0.005. **c** Immunoblot of indicated markers in MCF-7 or MCF-7^PAPP-A^ cells co-cultured with and without collagen. Scale bar 100 μm. **d** Representative images of 48 h Transwell in vitro invasion assays of MCF-7 and MCF-7^PAPP-A^ cells. Experiments repeated in technical triplicate. Scale bar 100 μm. Quantification is shown, unpaired *t* test with Welch’s correction. *p < 0.05. **e** Relative fold change of col1a1 mRNA transcript in MCF-7^PAPP-A^ over MCF-7 cells measured by real-time qPCR and normalized to actin. Mean + SEM, triplicate experiment. Unpaired *t* test with Welch’s correction. *p < 0.05. **f** Relative fold change of col1a1 mRNA transcript in MCF-7 cells treated in vitro with or without PAPP-A media for 24 h. PAPP-A media concentration is at 10 ng/mL of PAPP-A protein (quantified by ELISA). Measured by real-time qPCR and normalized to actin. Mean + SEM, triplicate experiment. Unpaired *t* test with Welch’s correction. **p < 0.005. **g** Immunoblot of LARP6 in MCF-7 cells treated in vitro with or without PAPP-A media for 24 h. PAPP-A media concentration is at 10 ng/mL of PAPP-A protein (quantified by ELISA). **h** Representative immunoblot of LARP6 in the mammary glands from non-transgenic or PAPP-A transgenic mice during virgin, involution, or late post-partum. *n* = 5 mice per group. **i** Immunoblot of rIGFBP-5 following a 3-h incubation in culture media from MCF-7 (CTL), MCF-7^PAPP-A^ (PA), or MCF-7 overexpressing proteolytic-dead mutant PAPP-A E483Q (E483Q). Immunoblot of PAPP-A secreted in culture media from MCF-7, MCF-7^PAPP-A^, and MCF-7^PAPP-A/E483Q^ cells. **j** Immunoblot of MCF-7 cells treated in vitro with culture media from MCF-7 (CTL), MCF-7^PAPP-A^ (PA), or MCF-7^PAPP-A/E483Q^ (E483Q) cells for 24 h. **k** Immunoblot of DDR2 and phospho-DDR2 in MCF-7 and MCF-7^PAPP-A^ cells treated in vitro with recombinant IGF-1 at 10 nM for indicated time points

A recent report described a novel signaling role for DDR2 in breast cancer metastasis through the stabilization of the epithelial to mesenchymal transition (EMT) transcription factor Snail (Fig. 3a) [39]. In addition to a critical role in activating EMT, Snail may also promote cancer stem-like subpopulations, drug resistance, and tumor cell invasion [42, 43]. Since we found that PAPP-A leads to an increase in collagen [22] (Fig. 1a, b) and promotes metastasis (Fig. 2j), we sought to address whether the DDR2/Snail axis of metastasis is active in PAPP-A-driven PABC (Fig. 3a).

To address this possibility, we performed immunohistochemistry of Snail on the mammary glands from non-transgenic or PAPP-A transgenic mice during involution or late post-partum. This analysis revealed a significant increase in nuclear expression of Snail, which has been reported to be the most aggressive form (Fig. 3b) [44, 45]. To confirm that the upregulation of Snail in cells overexpressing PAPP-A confers an increase invasion capacity, we first determine the level of Snail in MCF-7 and MCF-7^PAPPA^ cells co-cultured with and without collagen I to mimic the collagen-rich environment of involution and post-partum mammary glands. We found a significant elevation in Snail in the MCF-7^PAPP-A^ cells (Fig. 3c). While the levels of phosphorylated DDR2 (p-DDR2) could not be determined by immunohistochemistry due to the lack of anti-p-DDR2 antibody that recognizes mouse DDR2, we next aimed at determining the level of p-DDR2 in MCF-7 and MCF-7^PAPPA^ cells. MCF-7^PAPPA^ cells showed higher phosphorylated DDR2 (p-DDR2) and Snail expression co-cultured with and without collagen I (Fig. 3c). Therefore, these observations confirm that the DDR2/Snail axis is activated following the overexpression of PAPP-A. Since Snail is associated with invasion, we also measured the invasion capacity of MCF-7 and MCF-7^PAPPA^ cells using Transwell invasion assay. We found that MCF-7^PAPPA^ cells have a significantly higher cell invasive capacity (Fig. 3d).

Since we found that collagen is required in vivo for the activation of PAPP-A [17], we were surprised by the observation that in MCF-7^PAPP-A^ cells, DDR2 is activated in the absence of extraneous collagen. We hypothesized that this observation may be due to the ability of PAPP-A to promote the production of collagen in these cells. To test this possibility, we measured the level of collagen 1 mRNA in MCF-7 and MCF-7^PAPPA^ cells. We found an elevation in collagen mRNA expression MCF-7^PAPPA^ cells (Fig. 3e). Further, since we found that injecting PAPP-A promotes collagen deposition in vivo (Fig. 2c), we also measured collagen mRNA levels in MCF-7 cells treated with media containing PAPP-A. We found higher mRNA transcripts of col1a1 under these conditions (Fig. 3f).

La ribonucleoprotein domain family member 6 (LARP6) is a post-transcriptional modifier of col1a1 that enhances col1a1 transcripts’ translation by binding to a specific 5′ UTR stem-loop of col1a1 thereby both stabilizing col1a1 transcripts and aiding in their transport from the nucleus to the cytoplasm [46, 47]. IGF signaling has been previously reported to increase collagen production specifically by upregulating LARP6 [48, 49]. Therefore, since PAPP-A increases IGF signaling, we analyzed the level of LARP6 in MCF-7 cells treated with PAPP-A. We found that MCF-7 cells treated with PAPP-A resulted in an upregulation of LARP6 protein (Fig. 3g). To verify if this observation holds true in PAPP-A transgenic mice, we analyzed the level of LARP6 in non-transgenic and PAPP-A transgenic mammary glands during involution and in post-partum glands. We found that LARP6 protein is upregulated in vivo during involution but more significantly in post-partum glands (Fig. 3h). These results indicate that in addition to its known role in promoting IGF signaling, PAPP-A also promotes metastasis through the activation of DDR2 and promotes the stabilization of collagen through activation of LARP6.

PAPP-A is a large protease with multiple uncharacterized domains. Therefore, it remains possible that the action of PAPP-A on DDR2 is independent of its proteolytic function. To test this possibility, we treated MCF-7 cells with media containing wild-type PAPP-A and media containing a proteoloytic-dead mutant form of PAPP-A (E483Q). We confirmed that the E483Q mutant is inactive and unable to cleave recombinant IGFBP-5 in vitro (Fig. 3i). We found that wild-type but not inactive PAPP-A activates DDR2 and Snail in MCF-7 cells in vitro (Fig. 3j). Since PAPP-A activates IGF signaling, we then asked if IGF-1 downstream of PAPP-A is able to activate DDR2. To investigate this possibility, we tested the effect of exogenous IGF-1 on DDR2 in vitro. We found that IGF-1 activates DDR2 in MCF-7 cells, but this effect was markedly increased in MCF-7^PAPP-A^ cells (Fig. 3k). Further, MCF-7^PAPP-A^ cells expressing inactive PAPP-A also show some activation although the effect was markedly decreased compared to MCF-7^PAPP-A^ cells (Additional file 2: Figure S2).

These results suggest that through its ability to increase IGF signaling and the elevation in collagen, PAPP-A leads to the increased activation of DDR2 signaling.

### Loss of DDR2 abolishes the ability of PAPP-A to regulate cell invasion and Snail expression in vitro and in vivo

**Fig. 4:**
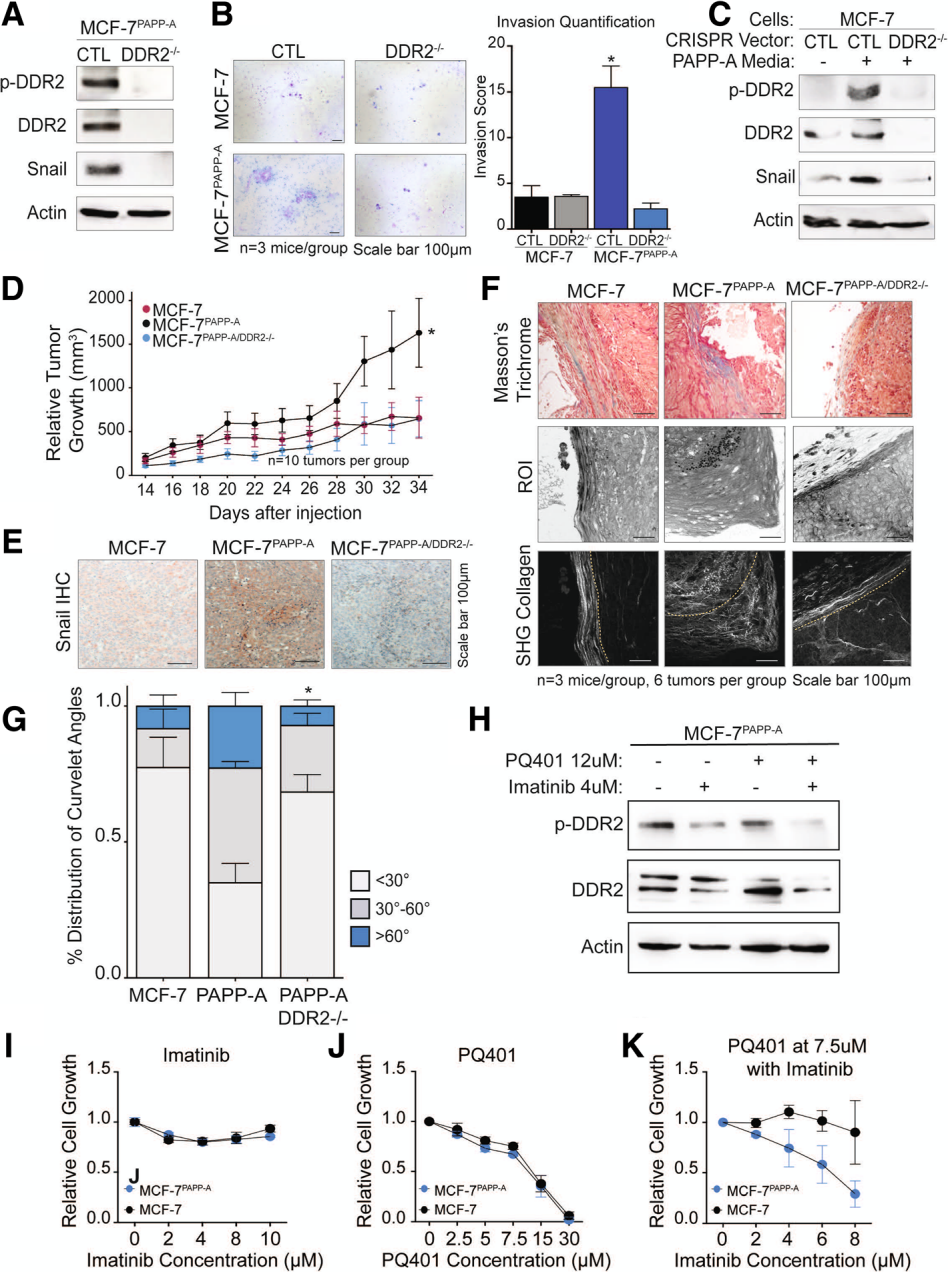
Loss of DDR2 abolishes cell invasion, Snail expression, and tumor growth by PAPP-A. **a** Immunoblot of indicated markers in MCF-7^PAPP-A^ CTL and MCF-7^PAPP-A^ DDR2^−/−^ cells. **b** Representative images of 48 h Transwell in vitro invasion assays of MCF-7 CTL, MCF-7 DDR2^−/−^, MCF-7^PAPP-A^ CTL, and MCF-7^PAPP-A^ DDR2^−/−^ cells. Experiments repeated in technical triplicate. Scale bar 100 μm. Quantification is shown, unpaired *t* test with Welch’s correction. *p < 0.05. **c** Immunoblot of indicated markers in MCF-7 CTL or MCF-7 DDR2^−/−^ cells treated with or without PAPP-A media for 24 h. **d** Relative tumor growth of MCF-7, MCF-7^PAPP-A^, and MCF-7^PAPP-A^ DDR2^−/−^ xenografts in a Matrigel-collagen mixture. *n* = 5 mice per group, ten tumors each. Each point represents the average of five mice, mean ± SEM. Unpaired *t* test at end point (MCF-7^PAPP-A^ vs. MCF-7^PAPP-A^ DDR2^−/−^). *p < 0.05 **e** Representative images of Snail IHC on MCF-7, MCF-7^PAPP-A^, and MCF-7^PAPP-A^ DDR2^−/−^ xenograft tumors in Matrigel-collagen mixture. *n* = 3 mice, six tumors total per group. Scale bar 100 μm. **f** Representative images of Masson’s trichrome collagen stain (blue) and second-harmonic generation (SHG) imaging of collagen (lower panels). Yellow dotted lines indicate the mammary tumor borders. Corresponding regions of interest (ROI) on magnified histological sections of MCF-7, MCF-7^PAPP-A^, or MCF-7^PAPP-A^ DDR2^−/−^ xenograft tumors in a Matrigel-collagen mixture. *n* = 3 mice per group. Scale bar 100 μm. **g** Quantification of TACS3 per total curvelets analyzed in MCF-7, MCF-7^PAPP-A^, or MCF-7^PAPP-A DDR2−/−^ xenograft tumors in a Matrigel-collagen mixture using the CurveAlign software. TACS3 characterized as curvelet angles 60–90 relative to the ductal border. *n* = 3 mice, six tumors total per group. Mean + SEM, unpaired *t* test with Welch’s correction (comparisons between TACS3 group only, MCF-7^PAPP-A^ vs. MCF-7^PAPP-A^ DDR2^−/−^). *p < 0.05. **h** Immunoblot of indicated markers in MCF-7^PAPP-A^ cells treated for 48 h at the indicated concentrations of PQ401 and imatinib. **i** Proliferation assay of MCF-7 and MCF-7^PAPP-A^ cells treated with imatinib at the indicated concentrations for 48 h. **j** Proliferation assay of MCF-7 and MCF-7^PAPP-A^ cells treated with PQ401 at the indicated concentrations for 48 h. **k** Proliferation assay of MCF-7 and MCF-7^PAPP-A^ cells treated with PQ401 at 7.5 μM in addition to the indicated concentrations of imatinib for 48 h

To further validate the role of DDR2 in the increased invasion of cells overexpressing PAPP-A, we used CRISPR to knock out DDR2 in MCF-7 and MCF-7^PAPP-A^ cells. First, we confirmed that DDR2 expression is abolished in the MCF-7^PAPPA-DDR2KO^ cells (Fig. 4a). Inhibition of DDR2 resulted in undetectable levels of Snail by immunoblot (Fig. 4a). Further, the decrease in Snail correlated with a marked decrease in cell invasive capacity (Fig. 4b). We confirmed that inhibition of DDR2 abolishes the activation of Snail by PAPP-A using MCF-7 cells and MCF-7 cells where DDR2 was inhibited by CRISPR (MCF-7^DDR2KO^) despite treatment with PAPP-A containing media (Fig. 4c).

Therefore, these results suggest that in addition to increasing IGF signaling and collagen production, PAPP-A leads to the activation of a distinct collagen signaling pathway mediated by DDR2 which promotes invasion.

To further explore the potential clinical relevance of DDR2 signaling in PAPP-A-driven PABC, we sought to investigate the effect of DDR2 in vivo by comparing the growth of MCF-7, MCF-7^PAPP-A^, and MCF-7^PAPP-A/DDR2KO^ xenografts in the mammary fat pads of nude mice. Additionally, we previously found that using a 1:1 Matrigel-collagen reduces the growth of MCF-7 xenografts compared to Matrigel alone [22]. This observation suggests that Matrigel-collagen mixture mimics the collagen-rich and anti-proliferative environment of a post-partum mammary gland [14, 22]. We therefore performed these xenografts using a 1:1 Matrigel-collagen mixture.

Consistent with our previous reports, we found that MCF-7^PAPP-A^ cells formed significantly larger tumor than MCF-7 cells under these conditions (Fig. 4d). However, this effect was abolished upon DDR2 depletion (Fig. 4d). Therefore, we conclude that DDR2 is a significant mediator of the accelerated growth of cells overexpressing PAPP-A in vivo.

Further, in agreement with the results obtained in vitro, we found that MCF-7^PAPP-A^ xenografts have the highest Snail protein expression by IHC, while MCF-7^PAPP-A/DDR2KO^ xenografts exhibit undetectable Snail expression (Fig. 4e).

We next analyzed the orientation of collagen in these xenografts. We found that MCF-7 xenografts exhibit elevated TACS1 but low TACS3, while MCF-7 ^PAPP-A^ xenografts show elevated TACS3 orientation (Fig. 4f, g). Strikingly, MCF-7^PAPP-A/DDR2KO^ xenografts had significantly less TACS3 regions of collagen and distribution of curvelet angles comparable to that seen in MCF-7 tumors (Fig. 4f, g). These results suggest a critical role for DDR2 in the ability of PAPP-A to promote and maintain collagen into a TACS3 orientation.

Imatinib has been shown to inhibit the activity of DDR2 [50, 51]. Since PAPP-A activates both IGF and DDR2 signaling, we tested the ability of the IGF inhibitor PQ401 alone or in combination with imatinib on the growth of MCF-7^PAPP-A^ cells. First, we confirmed that imatinib reduces the activation of DDR2 in these cells (Fig. 4h). However, the reduction was enhanced in cells treated with imatinib in combination with PQ410 (Fig. 4h). We found that while imatinib has no effect on the growth of either MCF-7 or MCF-7^PAPP-A^ cells (Fig. 4i) and PQ401 inhibited cell growth in both cell lines equally (Fig. 4j), the combination selectively inhibited the growth of the MCF-7^PAPP-A^ cells (Fig. 4k). Mechanistically, we found that MCF-7^PAPP-A^ cells have higher p-Akt levels than MCF-7 cells (Additional file 3: Figure S3). However, a decrease in p-Akt levels was only observed in MCF-7^PAPP-A^ cells when treated with the combination treatment of PQ401 and imatinib (Additional file 3: Figure S3). This observation is consistent with the observation that MCF-7^PAPP-A^ cells have higher DDR2 activation in response to treatment with IGF (Fig. 3k). Further, a recent study reported a positive crosstalk between DDR1 and IGF signaling, therefore raising the possibility that a similar crosstalk exists between DDR2 and IGF signaling [52].

These results indicate that complete inhibition achieved by either deletion of DDR2 by CRISPR or treatment with pharmacological combination is necessary to abolish the growth of the MCF-7^PAPP-A^ cells, while DDR2 inhibition by imatinib alone does not.

### Long lactation inhibits PAPP-A-driven TACS3 formation in post-partum mammary gland

**Fig. 5:**
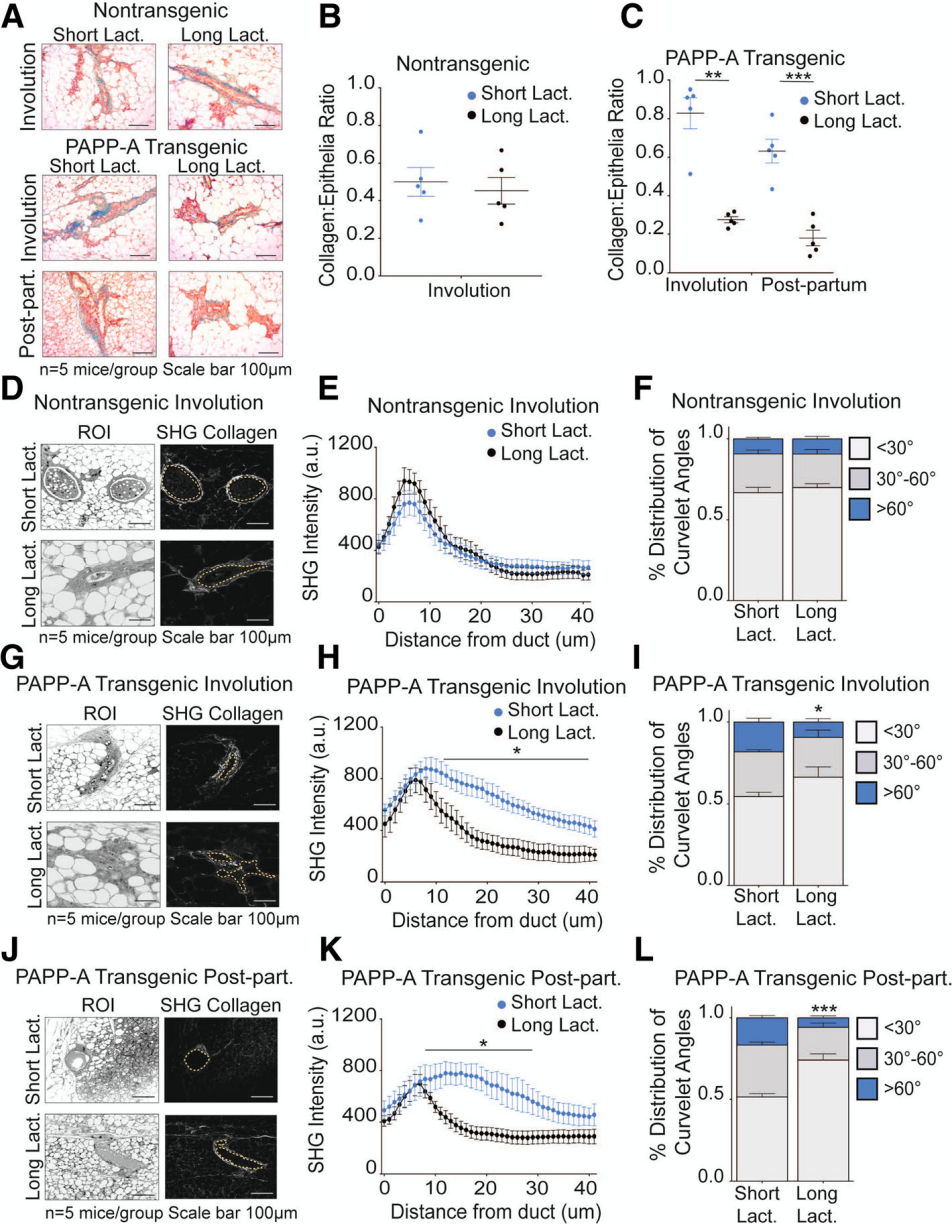
Long lactation inhibits PAPP-A-driven collagen deposition and TACS3 formation in post-partum mammary glands. **a** Representative images of Masson’s trichrome collagen stain (blue) on involuting or post-partum mammary glands from non-transgenic and PAPP-A transgenic mice following a short (2 days) or long (14 days) lactation period. *n* = 5 mice per group. All images from mice following short lactation are from the same experiment shown in Fig. 1a. Scale bar 100 μm. **b** Quantification of collagen per epithelial region by Masson’s trichrome stain on non-transgenic involuting mammary glands following a short (2 days) or long (14 days) lactation period from **a**. *n* = 5 mice, each point represents the average of ten ducts per mouse per time point. Mean ± SEM, unpaired *t* test with Welch’s correction. Analysis on short lactation samples is from the same analysis performed in Fig. 1b. **c** Quantification of collagen per epithelial region by Masson’s trichrome stain on PAPP-A transgenic involuting or post-partum mammary glands following a short (2 days) or long (14 days) lactation period from **a**. *n* = 5 mice, each point represents the average of ten ducts per mouse per time point. Mean ± SEM, unpaired *t* test with Welch’s correction: **p < 0.005, ***p < 0.0005. Analysis on short lactation samples is from the same analysis performed in Fig. 1c. **d** Representative second-harmonic generation (SHG) imaging of collagen on magnified ducts of histological sections of non-transgenic involuting mammary glands following a short (2 days) or long (14 days) lactation period (right panels) and corresponding regions of interest (ROI) (left panels) (*n* = 5 mice per group). All images of short lactation samples are from the same experiment shown in Fig. 1d. Scale bar 100 μm. **e** Graph of collagen intensity relative to the distance from the mammary duct borders of SHG images of non-transgenic involuting mammary glands following a short (2 days) or long (14 days) lactation period in **d**. Mean ± SEM. *n* = 5 mice per group, unpaired *t* test with Welch’s correction. Analysis on short lactation samples is from the same analysis performed in Fig. 1e. **f** Quantification of TACS3 per total curvelets analyzed in non-transgenic involuting mammary glands following a short (2 days) or long (14 days) lactation period using the CurveAlign software. TACS3 characterized as curvelet angles 60–90 relative to the ductal border. *n* = 5 mice per group. Mean + SEM, unpaired *t* test with Welch’s correction (comparisons between the TACS3 group only). Analysis on short lactation samples is from the same analysis performed in Fig. 1g. **g** Representative second-harmonic generation (SHG) imaging of collagen on magnified ducts of histological sections of PAPP-A transgenic involuting mammary glands following a short (2 days) or long (14 days) lactation period (right panels) and corresponding regions of interest (ROI) (left panels) (*n* = 5 mice per group). All images of short lactation samples are from the same experiment shown in Fig. 1d. Scale bar 100 μm. **h** Graph of collagen intensity relative to the distance from the mammary duct borders of SHG images of PAPP-A transgenic involuting mammary glands following a short (2 days) or long (14 days) lactation period in **e**. Mean ± SEM. *n* = 5 mice per group, unpaired *t* test with Welch’s correction. Analysis on short lactation samples is from the same analysis performed in Fig. 1f. **i** Quantification of TACS3 per total curvelets analyzed in PAPP-A transgenic involuting mammary glands following a short (2 days) or long (14 days) lactation period using the CurveAlign software. TACS3 characterized as curvelet angles 60–90 relative to the ductal border. *n* = 5 mice per group. Mean + SEM, unpaired *t* test with Welch’s correction (comparisons between the TACS3 group only). *p < 0.05. Analysis on short lactation samples is from the same analysis performed in Fig. 1g. **j** Representative second-harmonic generation (SHG) imaging of collagen on magnified ducts of histological sections of PAPP-A transgenic post-partum mammary glands following a short (2 days) or long (14 days) lactation period (right panels) and corresponding regions of interest (ROI) (left panels) (*n* = 5 mice per group). All images of short lactation samples are from the same experiment shown in Fig. 1d. **k** Graph of collagen intensity relative to the distance from the mammary duct edge of SHG images of PAPP-A transgenic post-partum mammary glands following a short (2 days) or long (14 days) lactation period in **f**. Mean ± SEM. *n* = 5 mice per group, unpaired *t* test with Welch’s correction: *p < 0.05. Analysis on short lactation samples is from the same analysis performed in Fig. 1f. **l** Quantification of TACS3 per total curvelets analyzed in PAPP-A transgenic post-partum mammary glands following a short (2 days) or long (14 days) lactation period using the CurveAlign software. TACS3 characterized as curvelet angles 60–90 relative to the ductal border. *n* = 5 mice per group. Mean + SEM, unpaired *t* test with Welch’s correction (comparisons between the TACS3 group only). *p < 0.05. Analysis on short lactation samples is from the same analysis performed in Fig. 1g

We previously reported that a long lactation of 2 weeks or more prior to involution inhibits the formation of PABC in the PAPP-A transgenic mice [22]. As long lactation represents an alternative method to inhibit the action of PAPP-A upstream of DDR2, we tested the effect of long lactation on the level of collagen deposition in post-partum glands. We harvested mammary glands from parous PAPP-A transgenic mice during involution (12 days post-weaning) or post-partum (2 months post-weaning) as described in Fig. 1 with the exception that mothers were kept with their litters for 2 weeks prior to the initiation of involution. These mammary glands were compared to the mammary glands from involuting and post-partum PAPP-A transgenic mice following a short lactation described in Fig. 1. In non-transgenic mice, the length of lactation had no effect as expected (Fig. 5a, b) and was therefore not pursued further. In PAPP-A transgenic mice, however, we found that long lactation significantly decreased collagen abundance in both involuting and post-partum mammary glands (Fig. 5a, b).

We next analyzed the orientation of collagen. Consistent with the lack of effect of long lactation on collagen abundance, we found that long lactation had no effect on collagen orientation in involuting mammary glands from non-transgenic mice (Fig. 5d–f). However, long lactation resulted in a distribution of collagen that is closer to the ductal border and decreased TACS3 in involuting mammary glands of PAPP-A transgenic mice (Fig. 5g–i). Importantly, the distribution of collagen and the frequency of TACS3 in PAPP-A transgenic involuting mammary glands after a long lactation mimic those observed in the non-transgenic involuting mammary glands (Fig. 5e, f).

In post-partum mammary glands from PAPP-A transgenic mice, we found that a long lactation also significantly altered collagen orientation, as indicated by a decreased presence of collagen dispersed from the ductal border and decreased TACS3 (Fig. 5j–l). Furthermore, the decrease in TACS3 in PAPP-A transgenic post-partum mammary glands following a long lactation was even more significant than that observed in involuting mammary glands after a long lactation (Fig. 5f, i, l). These results highlight that a longer lactation ablates the ability of PAPP-A to maintain an involution-like collagen in post-partum mammary glands.

### PAPP-A-driven signature correlates with distant metastasis in human breast cancers

**Fig. 6:**
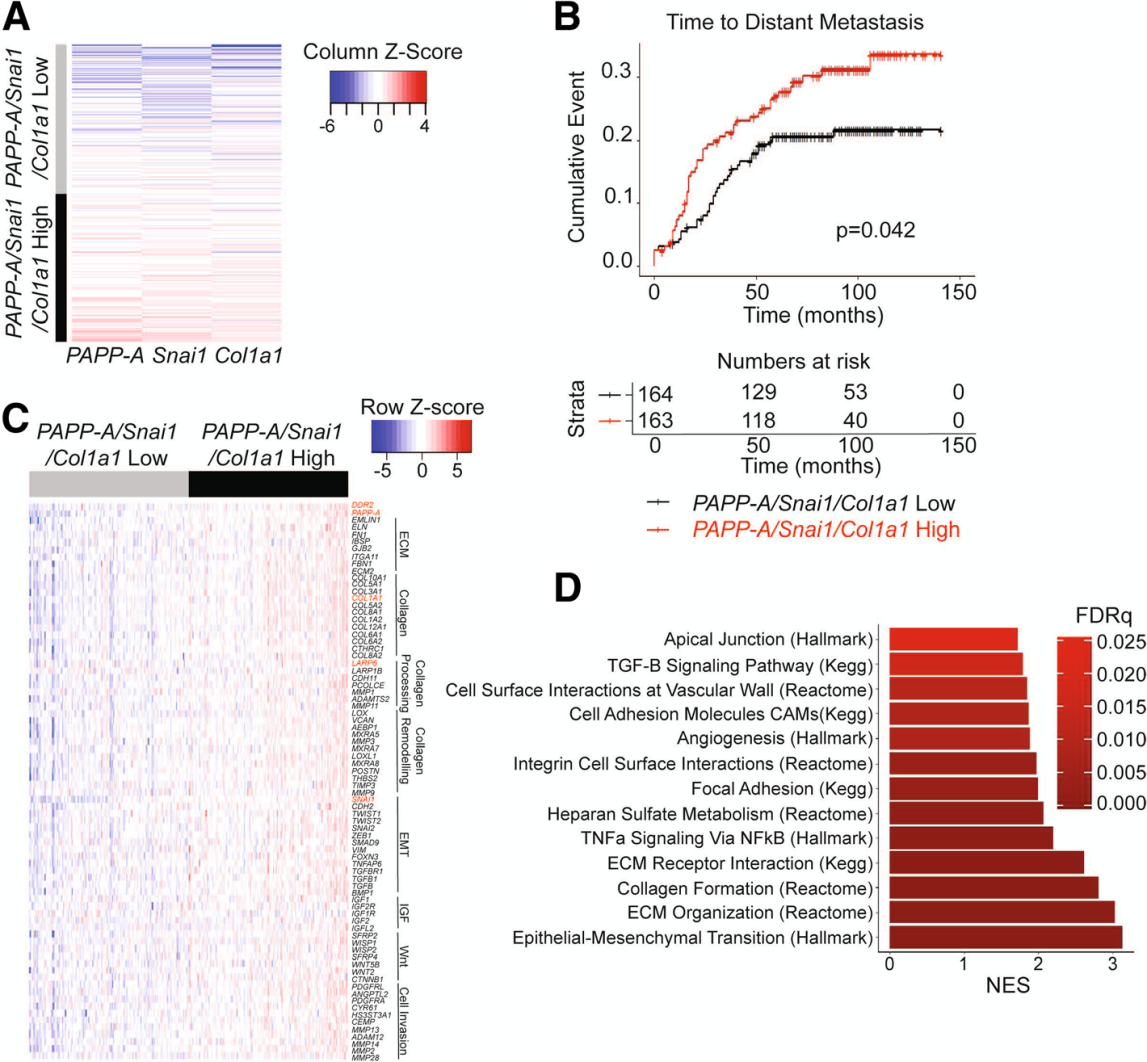
*PAPP-A/SNAI1/COL1A1* gene signature correlates with distant metastasis in breast cancer patients. **a** Heatmap of patients clustered based on their score for the *PAPP-A/SNAI1/COL1A1* signature (categorized according to the mean score value). Each row represents one patient, *n* = 327. **b** Kaplan-Meier curve for time to distant metastasis according to the *PAPP-A/SNAI1/COL1A1* score as defined in **a**. Number of patients at risk at each time point over a 150-month period is recorded below in table. **c** Heatmap representing the relative expression of a selected panel of 78 genes (FDR < .05) in the *PAPP-A/SNAI1/COL1A1*-low and *PAPP-A/SNAI1/COL1A1*-high patient populations. Genes are organized by pathways, as labeled adjacent to the gene names. The red text highlights relevant genes investigated in this study (*PAPP-A*, *DDR2*, *SNAI1*, *COL1A1*, and *LARP6*). Each column represents a patient, and each row represents a gene. **d** Chart of GSEA pathways significantly enriched in the *PAPP-A/SNAI1/COL1A1*-high patient population. Each bar represents the normalized enrichment score of the indicated pathway

Several barriers exist in the proper diagnosis of PABC. One is the lack of reliable diagnostic biomarkers. Despite the identification of PAPP-A as a potentially important biomarker of PABC, PAPP-A alone is inefficient as a biomarker since it is also overexpressed in the vast majority of breast cancer [21]. Our findings that virgin PAPP-A transgenic mice do not develop mammary tumors suggest that, despite its overexpression, PAPP-A does not act as an oncogene in the absence of collagen. Therefore, for PAPP-A to be useful as a diagnostic biomarker, it must be analyzed in the context of high collagen content. However, another difficulty arises from the fact that information regarding breastfeeding is either sparse or entirely missing from medical charts. Therefore, the use of combined expression of PAPP-A and collagen as a biomarker performs poorly as the impact of high expression of both genes can be ablated by long breastfeeding. Having identified the involvement of a DDR2/Snail axis in PAPP-A-driven PABC, we reasoned that Snail in combination with PAPP-A and collagen might represent a more reliable readout for monitoring the activation of the pathway. To test this hypothesis, we investigated the correlation between a three-gene signature of *PAPP-A*, *COL1A1*, and *SNAI1* and clinical outcomes in breast cancer patients. First, we generated a score based on the average gene expression of *PAPP-A/SNAI1/COL1A1* from a publicly available dataset of 327 patients with primary breast cancer (GSE20685) [27]. We next stratified patients based on this score into *PAPP-A*/*SNAI1/COL1A1* high and low (Fig. 6a). We found that the *PAPP-A*/*SNAI1/COL1A1*-high patient population develops distant metastases at a significantly higher rate than those patients with low values of the *PAPP-A*/*SNAI1/COL1A1* expression score (*P* = 0.042, Fig. 6b). This finding was validated in an additional dataset (GSE9893) with the *PAPP-A*/*SNAI1/COL1A1*-high patients developing distant metastases at a significantly higher rate (*p* = .0045, Additional file 4: Figure S4) [28].

To unravel the major molecular differences between patients with high and low values for our *PAPP-A*/*SNAI1/COL1A1* expression score, we next conducted differential gene expression between these patients [29]. We found 78 significantly upregulated genes of interest in the *PAPP-A*/*SNAI1/COL1A1*-high patient population (Fig. 6c, Additional file 5: Table S1). Of these 78 genes, highlighted in red is *PAPP-A*, *COL1A1*, and *SNAI1* as expected in addition to *DDR2* and *LARP6* (Fig. 6c). These 78 genes are involved in extracellular matrix components, collagen, collagen processing and remodeling, EMT, IGF signaling, Wnt signaling, and cell invasion and migration (Fig. 6c).

All significantly upregulated genes from the differential gene expression data were used for gene set expression analysis (GSEA) to identify significantly activated pathways in the *PAPP-A*/*SNAI1*/*COL1A1*-high patient population [30]. We found 13 statistically significant and relevant pathways active in the *PAPP-A*/*SNAI1/COL1A1*-high patient group (Fig. 6d, Additional file 6: Table S2). Of note, EMT, extracellular matrix organization, and collagen formation were the top 3 highly activated pathways in the *PAPP-A*/*SNAI1/COL1A1*-high patient group (Fig. 6d). This is consistent with the increased frequency of TACS3 collagen and cell invasion observed in our PAPP-A overexpressing cells in vitro and in vivo*.*

## Discussion

The existence of PABC as a distinct breast cancer subtype remains controversial. The major difficulties in the study of PABC are the lack of reliable biomarkers and the time frame after pregnancy of diagnosis of PABC. Currently, PABC is empirically defined as breast cancers arising 1–2 years after pregnancy despite the epidemiological findings that women who had children remain at higher risk of breast cancer for years compared to women who never had children [1]. Clearly distinguishing a PABC from a sporadic cancer that is unrelated to pregnancy in women diagnosed several years after their last pregnancy is a challenge.

Data presented in the current study support the provocative notion that the passage through a single pregnancy is sufficient to predispose a breast to the oncogenic action of PAPP-A. Our data imply that the acquisition of a mutation leading to PAPP-A overexpression at any point after pregnancy, even years, can hijack the high collagen abundance of a post-partum breast and convert this collagen into a TACS3 pro-tumorigenic collagen.

Considering that PAPP-A has been reported to be overexpressed in nearly all breast cancers [21] and that the majority of women worldwide have at least one pregnancy, the implication of our findings is that PAPP-A-driven PABC may be very frequent. If so, targeting PAPP-A for therapy may be an attractive therapeutic target. This possibility is supported by the fact that antibody against PAPP-A has been developed and shown to be effective in ovarian cancer [53]. Further, as genetic ablation of the PAPP-A gene leads to a reduction in several age-related pathologies and increases longevity by 30% [54], the prediction is that anti-PAPP-A therapy, in marked contrast to current therapies, would have limited side effects and may actually improve general health.

Further, considering the elevated rate of PAPP-A overexpression in breast cancer, targeting PAPP-A in the prevention setting also appears as a potential avenue of future research. In this regard, extended breastfeeding has been shown to have a protective effect against breast cancer [55]. Our results suggest that the inhibition of PAPP-A by its inhibitors stanniocalcins 1 and 2 (STC1, STC2) during lactation may play an important role in the protective effect of breastfeeding. Mechanistically, we reported that the protective effect of lactation is due to the production of STC1 and STC2 by the ovaries during lactation [22, 56, 57]. Our results proposed a model whereby long lactation allows for the saturation of PAPP-A by STC1 and 2 such that upon initiation of involution, PAPP-A remains inactive and cannot cleave IGFBP-5 [22]. As a result, the pro-tumorigenic cascade initiated by PAPP-A is prevented [22].

**Fig. 7:**
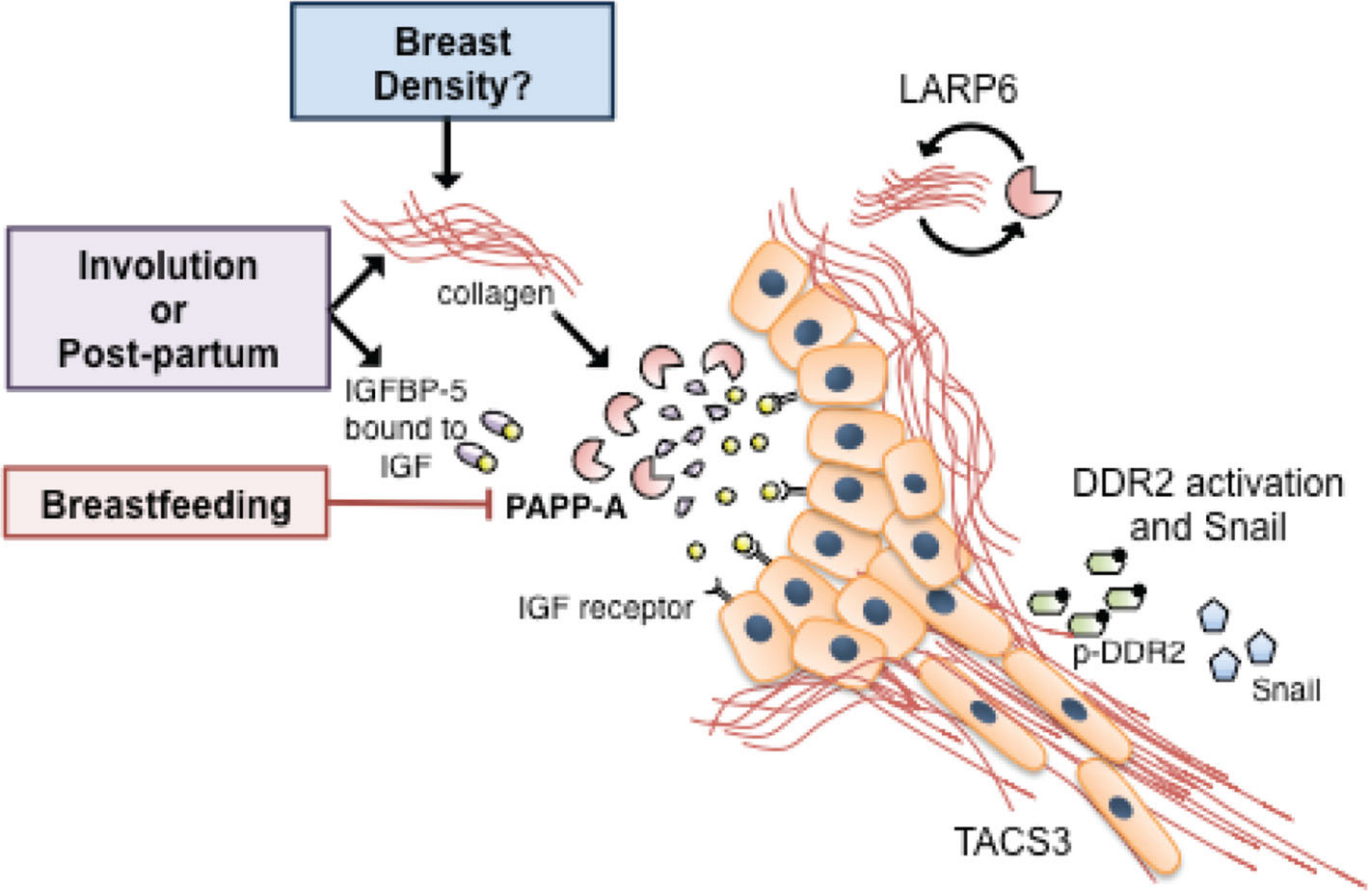
Model of PAPP-A-driven PABC. Schematic of our current understanding of PAPP-A-driven PABC, where red boxes indicate factors that have been established to activate the pathway, blue boxes indicate factors inhibiting the pathway, and green boxes factors that are hypothesized to affect the pathway. In this model, elevated content in collagen is necessary for PAPP-A to cleave IGFBP-5 in the mammary gland. The elevated level of collagen can be provided through distinct avenues including involution and, as demonstrated in the current study, post-partum environment. We postulate that high breast density may contribute the activity of PAPP-A. Conversely, extended breastfeeding appears to inhibit PAPP-A. Upon activation of PAPP-A and cleavage of IGFBP-5, free IGFs are released and able to bind and activate the IGF receptor, which results in increased IGF signaling, increased collagen deposition, and extended involution, in the case of an involuting mammary gland. We show that LARP6 is activated following PAPP-A overexpression and as a chaperone of the mRNA of collagen, LARP6 contributes to the elevation in collagen deposition in PAPP-A-driven mammary tumors. In addition, the collagen receptor DDR2 is activated, which leads to the DDR2/Snail axis of metastasis reported by others. In a mechanism that remains unclear, DDR2 promotes the formation of TACS3 collagen, which facilitates metastasis

Our findings that collagen is an important co-factor that contributes to the oncogenic activity of PAPP-A also raises the question of the potential link between PAPP-A and breast density. Mammographic density is one of the best predictors of breast cancer risk [58]. One prediction is that women with dense breasts, which have a higher content of collagen, maybe more prone to PAPP-A-driven PABC. In addition, a recent GWAS have shown that low IGFBP-5 is linked to increased risk of breast cancer [26]. This observation is also consistent with an important role of PAPP-A in breast cancer, since breasts with low endogenous level of IGFBP-5 are likely to be more sensitive to the oncogenic action of PAPP-A since the high PAPP-A/IGFBP-5 ratio would favor a more effective elimination of IGFBP-5. Therefore, the results of our study suggest that in the context of PAPP-A-driven PABC, breast density, SNPs in IGFBP-5, and history of breastfeeding must all be considered (Fig. 7).

Collectively, therefore, combining breast density, SNPs that reduce expression of IGFBP-5, pregnancy, and breastfeeding history holds the potential for the development of a predictive test for risk of breast cancer. Currently, genetic testing for the risk of breast cancer is limited to the sequencing of BRCA1 gene for women with a history of breast cancer. This possibility will be tested in the future.

In addition to offering a potentially important link between several known factors of breast cancer risk, our current study also identifies novel functions of PAPP-A, namely the elevation of collagen through LARP6 [46, 47] and the activation of the collagen receptor DDR2. We propose a model whereby both the crosstalk between the high IGF signaling by proteolytic cleavage of IGFBP-5 and increasing collagen deposition synergizes to activate DDR2 [22, 52, 59]. Further, we show that the resulting activation of DDR2 leads to an increase in Snail and cell invasive capacity. The effects of PAPP-A on invasion are significant as PABC is typically a more aggressive form of breast cancer, characterized by higher rates of recurrence, metastasis, and poorer patient survival [1, 4, 60, 61]. This finding is also consistent with the observation that PABC exhibit an increased rate of triple-negative breast cancer, which have the highest rate of mutation in p53 and we have previously shown mutant p53 activates the transcription of PAPP-A [1, 33, 62, 63]. We show here that PAPP-A promotes these physiological effects through TACS3 maintenance and DDR2/Snail signaling axis of invasion. How TACS3 is formed remains a mystery, but it has been reported that inhibition of DDR2 abolishes its formation [39].

We show that inhibition of DDR2 by CRISPR abolishes the ability of PAPPA to promote invasion. The role of DDR2 in PAPP-A-driven PABC adds to the growing evidence of the importance of DDR2 in breast cancer metastasis and that it is active in both tumor and stromal cells [40]. Our findings suggest the possibility of DDR2 as a novel therapeutic target in PAPP-A-driven PABC. A summary of how LARP6 and DDR2 add to our current understanding of PAPP-A-driven PABC is shown in Fig. 7.

## Conclusions

Despite extensive research in breast cancer and the fact that pregnancy and breastfeeding are the fundamental roles of breasts, history of pregnancy and breastfeeding is not included in standard clinical charts nor considered in diagnosed patients. In addition, pregnancy-associated breast cancer is empirically defined as those arising within 2 years after pregnancy and therefore misconceived as being rare. In fact, a recent meta-analysis combining the results of 15 studies confirmed that women remain at higher risk of breast cancer following pregnancy for more than 20 years, suggesting that healthcare providers should consider parity as a risk factor [6]. The results presented here challenge the current definition of PABC and support the conclusions of this recent study. Our data suggest that parity imposes a long-term predisposition to PAPP-A-driven breast cancers, a notion that much better reflects the epidemiological findings [1–3, 6]. Further, the data presented in the current study strongly argue that information regarding pregnancy and lactation history should be mandatory in medical charts of breast cancer patients. Our results suggest that this information, combined with the analysis of collagen and the *PAPP-A* genetic signature, holds the potential to identify patients at higher risk of recurrence. Further, the current study highlights the potential therapeutic interventions. Therefore, the translational impact spans from the need for basic information on medical charts to the development of a diagnostic signature and targeted intervention.

## Additional files

Additional file 1: **Figure S1.** PAPP-A media injections do not affect collagen abundance in virgin mammary glands. a) Representative images of Masson’s trichrome collagen stain (blue) on non-transgenic virgin mammary glands treated with control or PAPP-A injections. *n* = 3 mice per group. Scale bar 100 μm. b) Quantification of collagen per epithelial region by Masson’s trichrome stain on non-transgenic virgin mammary glands treated with control or PAPP-A injections. *n* = 3 mice per group, each point represents the average of ten ducts per mouse per group. Mean ± SEM, unpaired *t* test with Welch’s correction. (PDF 232 kb)

Additional file 2: **Figure S2.** Mutant PAPP-A decreases DDR2 activation by IGF-1: a) immunoblot of DDR2 and phospho-DDR2 in MCF-7^PAPP-A^ and MCF-7^PAPP-A-E483Q^ cells treated with recombinant 10 nM IGF-1 at indicated time points. (PDF 139 kb)

Additional file 3: **Figure S3.** Combination of PQ401 and imatinib treatment blocks p-Akt in vitro: a) immunoblot of the indicated markers in MCF-7 and MCF-7^PAPP-A^ cells treated at the indicated concentrations of PQ401 and imatinib. (PDF 157 kb)

Additional file 4: **Figure S4.** Secondary validation of time to distant metastasis: a) Kaplan-Meier curve for time to distant metastasis according to the *PAPP-A/SNAI1/COL1A1* score. Number of patients at risk at each time point over a 160-month period is recorded below in table. (PDF 162 kb)

Additional file 5: **Table S1.** LIMMA differential gene expression analysis *PAPP-A/SNAI1/COL1A1* gene signature: complete data on differential gene expression analysis on *PAPP-A/SNAI1/COL1A1*-high vs. *PAPP-A/SNAI1/COL1A1*-low patient groups. Each row is one gene (indicated by each probe used), and each column is as labeled. (XLS 9760 kb)

Additional file 6: **Table S2.** GenePattern gene set expression analysis (GSEA) *PAPP-A/SNAI1/COL1A1* gene signature: complete data on GSEA in *PAPP-A/SNAI1/COL1A1*-low patient group. Data represents the downregulated pathways in the *PAPP-A/SNAI1/COL1A1*-low patient group. Each row is one gene ontology term, and each column is as labeled. (XLS 64 kb)

## Abbreviations

Col1a1: Collagen type 1
DDR2: Discoidin domain receptor 2
EMT: Epithelial-mesenchymal transition
IGF: Insulin-like growth factor
IGFBP-5: Insulin-like growth factor binding protein-5
LARP6: La ribonucleoprotein 6
PABC: Pregnancy-associated breast cancer
PAPP-A: Pregnancy-associated plasma protein A
SHG: Second-harmonic generation
Snai1: Snail
STC: Stanniocalcin
TACS: Tumor-associated collagen signature
