# Supplementary figures and images for: Parity predisposes breasts to the oncogenic action of PAPP-A and activation of the collagen receptor DDR2
Source: Breast Cancer Res. 2019 May 2;21:56. doi: 10.1186/s13058-019-1142-z (PMC6498606; doi:10.1186/s13058-019-1142-z)

Supplemental Figure 1

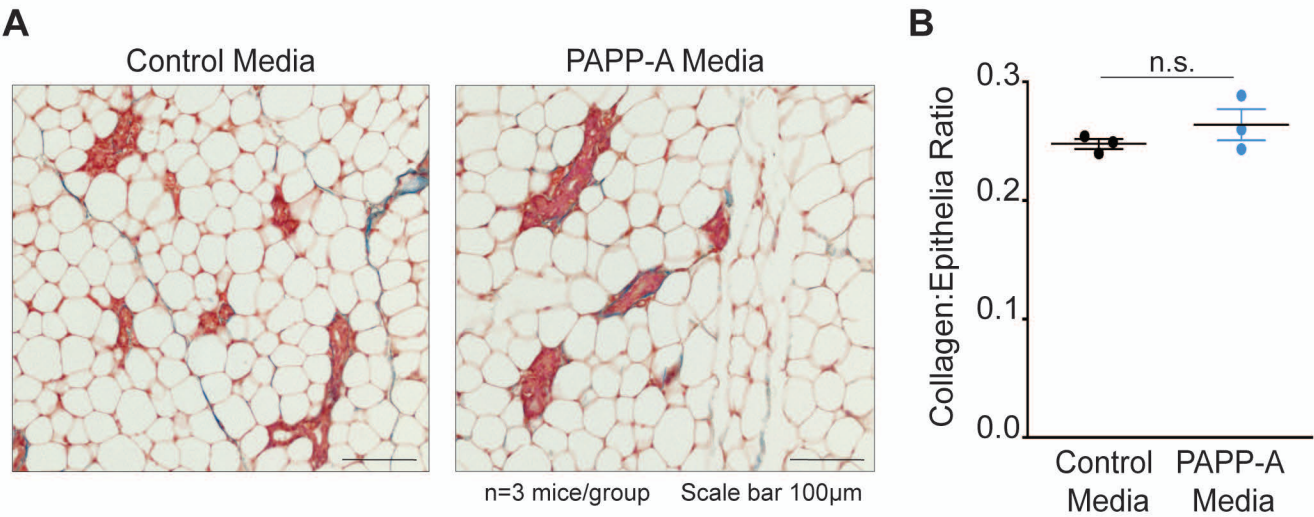

Supplement: Supplementary file 1 — Figure S1. PAPP-A media injections do not affect collagen abundance in virgin mammary glands. a) Representative images of Masson’s trichrome collagen stain (blue) on non-transgenic virgin mammary glands treated with control or PAPP-A injections. n = 3 mice per group. Scale bar 100 μm. b) Quantification of collagen per epithelial region by Masson’s trichrome stain on non-transgenic virgin mammary glands treated with control or PAPP-A injections. n = 3 mice per group, each point represents the average of ten ducts per mouse per group. Mean ± SEM, unpaired t test with Welch’s correction. (PDF 232 kb) [file 13058_2019_1142_MOESM1_ESM.pdf]

Supplemental Figure 2

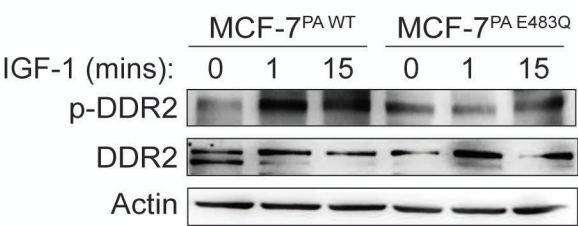

Supplement: Supplementary file 2 — Figure S2. Mutant PAPP-A decreases DDR2 activation by IGF-1: a) immunoblot of DDR2 and phospho-DDR2 in MCF-7PAPP-A and MCF-7PAPP-A-E483Q cells treated with recombinant 10 nM IGF-1 at indicated time points. (PDF 139 kb) [file 13058_2019_1142_MOESM2_ESM.pdf]

Supplemental Figure 3

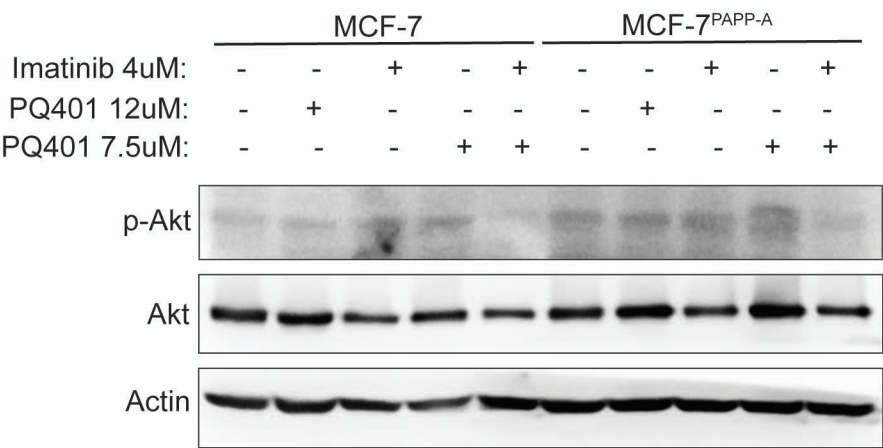

Supplement: Supplementary file 3 — Figure S3. Combination of PQ401 and imatinib treatment blocks p-Akt in vitro: a) immunoblot of the indicated markers in MCF-7 and MCF-7PAPP-A cells treated at the indicated concentrations of PQ401 and imatinib. (PDF 157 kb) [file 13058_2019_1142_MOESM3_ESM.pdf]

Supplemental Figure 4

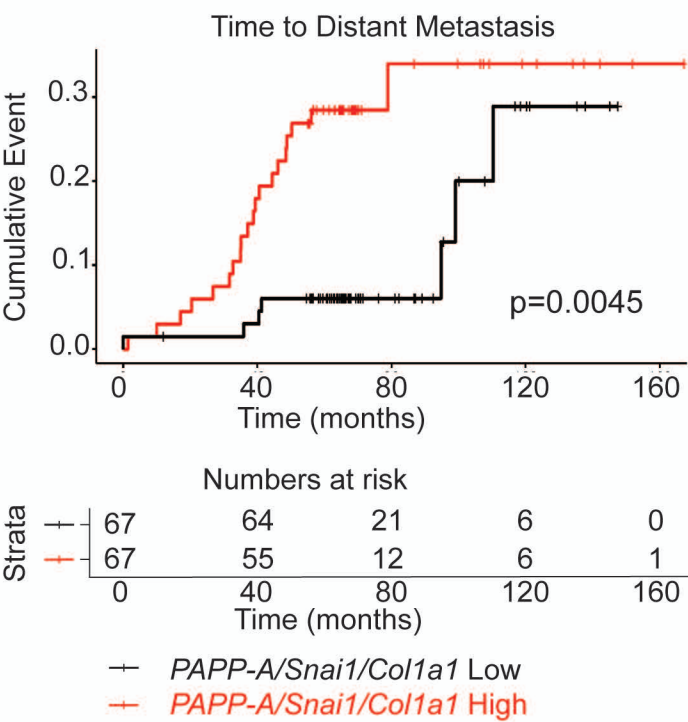

Supplement: Supplementary file 4 — Figure S4. Secondary validation of time to distant metastasis: a) Kaplan-Meier curve for time to distant metastasis according to the PAPP-A/SNAI1/COL1A1 score. Number of patients at risk at each time point over a 160-month period is recorded below in table. (PDF 162 kb) [file 13058_2019_1142_MOESM4_ESM.pdf]
